# Common reef-building coral in the Northern Red Sea resistant to elevated temperature and acidification

**DOI:** 10.1098/rsos.170038

**Published:** 2017-05-17

**Authors:** Thomas Krueger, Noa Horwitz, Julia Bodin, Maria-Evangelia Giovani, Stéphane Escrig, Anders Meibom, Maoz Fine

**Affiliations:** 1 Laboratory for Biological Geochemistry, School of Architecture, Civil and Environmental Engineering, Ecole Polytechnique Fédérale de Lausanne (EPFL), 1015 Lausanne, Switzerland; 2 The Mina and Everard Goodman Faculty of Life Sciences, Bar-Ilan University, Ramat-Gan 52900, Israel; 3 The Interuniversity Institute for Marine Sciences, Eilat 88103, Israel; 4 Center for Advanced Surface Analysis, Institute of Earth Sciences, University of Lausanne, 1015 Lausanne, Switzerland

**Keywords:** global climate change, coral bleaching, *Stylophora pistillata*, *Symbiodinium*, NanoSIMS, coral refugia

## Abstract

Coral reefs are currently experiencing substantial ecological impoverishment as a result of anthropogenic stressors, and the majority of reefs are facing immediate risk. Increasing ocean surface temperatures induce frequent coral mass bleaching events—the breakdown of the nutritional photo-symbiosis with intracellular algae (genus: *Symbiodinium*). Here, we report that *Stylophora pistillata* from a highly diverse reef in the Gulf of Aqaba showed no signs of bleaching despite spending 1.5 months at 1–2°C above their long-term summer maximum (amounting to 11 degree heating weeks) and a seawater pH of 7.8. Instead, their symbiotic dinoflagellates exhibited improved photochemistry, higher pigmentation and a doubling in net oxygen production, leading to a 51% increase in primary productivity. Nanoscale secondary ion mass spectrometry imaging revealed subtle cellular-level shifts in carbon and nitrogen metabolism under elevated temperatures, but overall host and symbiont biomass proxies were not significantly affected. Now living well below their thermal threshold in the Gulf of Aqaba, these corals have been evolutionarily selected for heat tolerance during their migration through the warm Southern Red Sea after the last ice age. This may allow them to withstand future warming for a longer period of time, provided that successful environmental conservation measures are enacted across national boundaries in the region.

## 1. Introduction

Coral reefs are among the most diverse and productive ecosystems on the planet. Their carbonate structures are produced by scleractinian hermatypic corals that live in association with a range of micro- and macroscopic organisms [1]. Most reef-building corals harbour a high density of photosynthesizing, endosymbiotic microalgal dinoflagellates of the genus *Symbiodinium*. These symbionts are essential because they transfer assimilated inorganic nutrients to the coral host, complementing an otherwise heterotrophic lifestyle, and provide the coral with a substantial ecological advantage in generally nutrient-poor tropical oceans [2]. This symbiosis is, however, sensitive to environmental changes such as sea surface temperature (SST) variations. In most reef locations around the world, corals live close to their upper thermal threshold and are thus vulnerable to the continuing warming of the upper ocean, which triggers widespread bleaching [3,4]. Many previously pristine reef systems (e.g. in the Caribbean) are now degraded due to the combined impact of local stressors and ocean warming [5–9]. Indeed, the frail current state of many existing coral reefs and the predicted further decline in live cover and ecosystem functioning as a result of human activity raise doubt whether coral reefs will survive the twenty-first century [10,11]. Assessing reef resilience and testing the ability of individual coral species to cope with the expected warming and acidification of tropical oceans is therefore of major importance. Within this context, identifying resistant coral species and/or geographical reef refugia has now become a scientific necessity.

Given the global distribution of corals and the mosaic nature of the coral holobiont (the symbiotic unit composed of microalgae, bacterial community and the animal host), much research has been devoted to establishing the acclimation potential of individual corals and the adaptation of reef communities in locations with extreme environmental settings. The presence of corals in seasonal hot water locations (e.g. the Southern Red Sea, Persian Gulf) as well as locations with large diel temperature fluctuations (reef flat, lagoons, e.g. Ofu, Samoa) demonstrates that corals have the potential to cope with elevated ocean temperatures [12,13]. Nevertheless, most tropical corals live close to their upper thermal threshold and their symbiotic relationship typically breaks down when exposed to 1–2°C above their local summer maximum for a month or more [14], although absolute bleaching thresholds can be as low as 27°C in Rapa Nui or as high as 35°C in the Persian Gulf [15]. Building on these findings, research employing large-scale mesocosms to manipulate seawater conditions has investigated whether and how thermal pre-conditioning alters stress performance as well as the traits of subsequent coral generations [16–19]. Enhancing environmental tolerance to temperature and pH through assisted evolution and selective breeding of coral species might substantially improve reef restoration projects of any scale [17].

**Figure 1.**
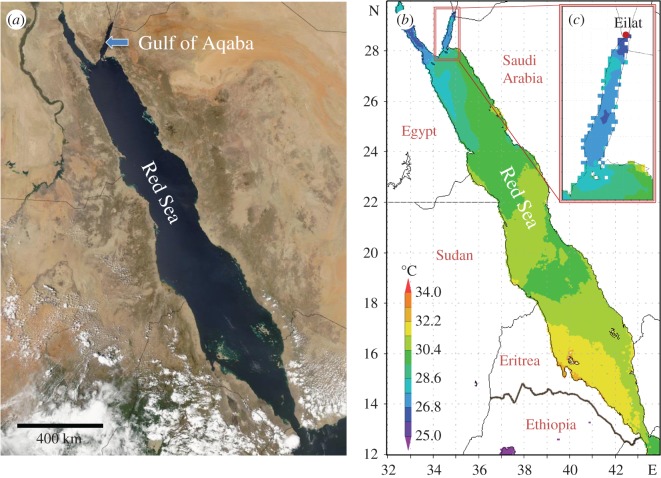
Geographical location of the Gulf of Aqaba (*a*) and thermal profiles of the Red Sea (*b*) and study area (Eilat, Israel) (*c*). Representative average sea surface temperatures (SSTs) in summer (July–Sep 2014) in the central and southern Red Sea are much higher than in the Gulf of Aqaba. The long-term August maximum at the study location is 26.67 ± 0.75°C (1988–2016), and daily mean SSTs usually do not exceed 28°C (with the exception of 2007 and 2012; figure 2 and electronic supplementary material, figure S1*d*).

**Figure 2.**
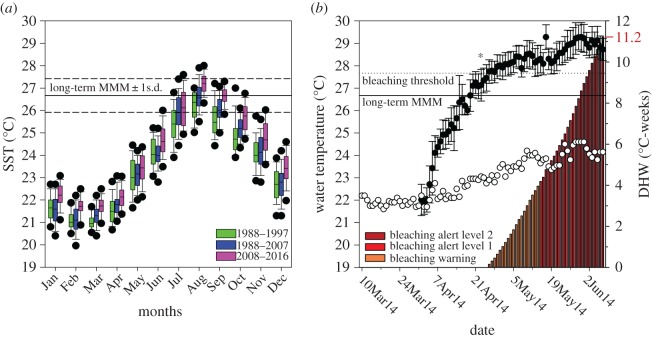
Long-term temperature record for GoA (Eilat area) and experimental thermal profile. (*a*) Monthly SST variations in the Eilat area based on daily SST records shown in decadal intervals from the period 1988–2016. The long-term maximum monthly mean (MMM) ± 1 s.d. is indicated. Black dots in boxplots show the 5th and 95th percentiles. (*b*) Experimental thermal profile for ambient (white) and elevated (black) temperature treatment (circles, left axis). Asterisk indicates the point when the pH was lowered to 7.8. Long-term MMM (solid; 1988–2016) and bleaching threshold (MMM+1°C, dashed) are indicated. Cumulative heat stress expressed as degree heating weeks (DHWs) are shown as bars (right axis). Indicated NOAA coral reef watch definitions correspond to possible bleaching (orange), likely bleaching (red) and likely mortality (dark red) [21].

Besides these experimental efforts and objectives, there are locations that provide a natural setting for testing general questions with regard to coral adaptation and physiological performance under environmental change. The Red Sea represents such a habitat. Here, the present coral population naturally underwent an evolutionary selection for thermal tolerance as the Red Sea was recolonized through the hot waters in the South after the last glacial cycle, 6000–7000 years ago [20]. The northward migration and larval dispersal through this thermal bottleneck positively selected for thermal robustness, enabling present populations in the Central and Southern Red Sea to live in waters with SSTs above 30°C in summer (figure 1). The further colonization of the Northern end of the Red Sea, however, led to a situation where heat-selected populations encountered cooler waters. Fringing reefs in the Gulf of Aqaba (GoA) (electronic supplementary material, figure S1*a*) experience much lower water temperatures with a long-term mean summer SST of only 26.2 ± 0.9°C (mean ± s.d.; Jul, Aug, Sep; 1988–2016) at the Northern tip in the Eilat/Aqaba area (figures 1 and 2; electronic supplementary material, figure S1*d*; [22]). Based on the observation that the GoA summer SST has risen by 0.4–0.5°C per decade over the last 30 years (figure 2; electronic supplementary material, figure S1*d,e*; [23]; www.coastalwarming.com: Point IDs 21_14968, 21_14765, 21_14766), these corals will experience summer temperatures 1–2°C above their current long-term maximum monthly mean (MMM) of 26.67 ± 0.75°C (August; 1988–2016) in the second half of the century. The unique evolutionary history of modern Red Sea corals raises the question of whether the present coral community in the GoA has the potential to better cope with ocean warming, considering that they start their warming trajectory from a lower summer SST and descended from a heat-selected founder population.

Here we test this hypothesis by documenting how the scleractinian coral *Stylophora pistillata* [24] (electronic supplementary material, figure S1*b*) from the Northern part of the GoA responds physiologically to an extended period of elevated SST and reduced pH. *S. pistillata* is a cosmopolitan species that is prominent in the Red Sea, includes multiple genetic host clades that exist in different thermal environments around the world and has been extensively used for all levels of physiological coral research [25]. Considered an r-strategist [26], this species is expected to be sensitive to environmental disturbances. We tested how essential physiological processes are affected by the isolated and combined effects of elevated temperature (six weeks with 1.2–2.4°C above long-term MMM) and lowered pH (−0.3 units; worst case projection for oceanic surface pH in tropical regions under IPCC scenario RCP8.5 [27]). We monitored a broad suite of physiological functions including photosynthesis, symbiont carbon/nitrogen fixation and translocation, protein, carbohydrate and antioxidant metabolism of both symbiotic partners, as well as calcification and holobiont appearance (symbiont density and pigmentation).

## 2. Material and methods

### 2.1. Long-term temperature data record and visualizations

Long-term temperature records were derived from manual measurements taken by Prof. Amatzia Genin (IUI Eilat) between 1988 and 2011 [20] and hourly SST records for the Eilat area (01/01/2007–14/11/2016, *N* = 86383) from the Israel National Monitoring Program (NMP) [22], reduced to daily mean values (electronic supplementary material, figure S1*d*). Comparing both datasets for an overlapping period (2007–2011) confirms that the values of the Genin dataset are within −0.21°C (10% quantile) and +0.27°C (90% quantile) of the daily mean of the NMP data (*N* = 1826). Visualizations of surface temperature data for the Red Sea were done using the Giovanni online data system, developed and maintained by the NASA Goddard Earth Sciences Data and Information Services Center (GES DISC).

### 2.2. Collection and maintenance of corals

All experiments were conducted using the outdoor Red Sea Simulator aquarium set-up at the Interuniversity Institute (IUI) for Marine Sciences in Eilat, Israel, between March and June 2014. Nine different *Stylophora pistillata* colonies (replicate ID A--I) were collected from the coral nursery of the IUI (4–8 m depth) that contains large colonies collected over time from different locations along the fringing reefs of Eilat (Gulf of Aqaba; electronic supplementary material, figure S1*a*). Corals were fragmented (4–8 cm pieces) and acclimated to the aquarium set-up (starting 10 March 2014) in 35 l flow-through aquaria (>60 l h^−1^) with ambient seawater under a shading tent in the aquaria array (electronic supplementary material, figures S1*c* and S2). Light levels were approximately one-fifth of ambient photosynthetically active radiation (PAR; approx. 300–400 µmol m^−2^ s^−1^ at midday; electronic supplementary material, figure S3*c*) with daylight length of approximately 12–14 h for the experimental period. Small aquaria pumps close to the water surface provided additional flow while breaking the surface to increase light scattering.

### 2.3. Experimental treatments and sampling

Temperature and pH (two levels per factor) were manipulated in the form of a full factorial design with three replicate aquaria (I, II, III) per treatment (electronic supplementary material, figure S2). Each replicate aquarium contained three of the nine coral colonies (A--I) and these replicate triplets were consistent across all treatments (i.e. I-ABC, II-DEF, III-GHI). Different sets of aquaria were supplied with seawater from four large seawater reservoirs (2000 l each, with water pumped in from a 30 m depth) of which two acted as mixing tanks for CO_2_-bubbling to lower the seawater pH (electronic supplementary material, figure S2). Temperature was controlled individually by heat exchangers in heated aquaria. The process of assigning collected coral colonies to replicate triplets and triplets to treatment aquaria was completely randomized. Twelve treatment aquaria were randomly chosen (within the subsets of heated and control aquaria) from 24 available aquaria of the Red Sea Simulator system (#109–120; #209–220; electronic supplementary material, figure S2). Note that this experiment represents a randomized set-up, but replicates are interdependent within the pH treatment factor (*sensu* B-4 design; [28]) and independent pH treatment replication is thus *N* = 2. However, we demonstrate the absence of mixing-tank effects in the low pH treatment by testing main physiological variables of the DEF triplet (chosen randomly; mixing tank 1 supply) against the GHI triplet (mixing tank 2 supply) in a balanced two-way ANOVA (cf. electronic supplementary material, figure S2 and table S6). Water temperature and pH were constantly monitored in each aquarium by a remotely operating robot (electronic supplementary material, figure S1*c* and S3).

On 1 April 2014, after 22 days of coral acclimation to the aquarium system, water temperature was gradually increased in the elevated temperature treatment to +5°C above ambient conditions. Once daily treatment temperature variation had completely crossed the long-term MMM for surface water in the Gulf of Aqaba, the ambient pH of approximately 8.1 was lowered to approximately 7.8 in the respective treatment aquaria (22 April 2014).

All physiological proxies were assessed after 47 days of exposure to combined temperature and pH stress. Cumulative heat stress, expressed as degree heating week (DHW; [21]), amounted to 11.2 DHW and the final treatment temperature in the 10 days leading up to the measurements was 29.1 ± 0.2°C (mean ± s.d.), i.e. 2.2°C above the MMM and approximately 5°C above the ambient temperature (figure 2). Due to the size of the experiment and time demand for live measurements of photosynthesis and respiration, sampling was performed over 3 consecutive days, measuring the first, second and third replicate aquarium of each treatment, respectively. Coral fragments were returned to their respective aquaria after chlorophyll fluorometry and respiratory measurements, to be snap-frozen in liquid nitrogen on the following day. This protocol allowed equal measurement conditions for all fragments, while minimizing the potential immediate effects that all live measurements had on the biochemical variables**.** All presented physiological data were taken on 5–7 June 2014. An isotopic labelling ([^13^C]-bicarbonate and [^15^N]-nitrate) experiment, with subsequent nanoscale secondary ion mass spectrometry (NanoSIMS) and electron microscopy (EM) imaging, was performed on 8 June 2014.

### 2.4. Pulse amplitude modulation fluorometry

Coral fragments were dark-acclimated for 20 min in their respective treatment and rapid light curves were generated (RLC, 0–701 µmol m^−2^ s^−1^ PAR, 20 s intervals), using an Imaging-PAM fluorometer (MI 3, SI 10, gain 2, damp 2, saturating width 0.8 s; Heinz Walz GmbH, Effeltrich, Germany). All pulse amplitude modulation (PAM) measurements were conducted between 8.00 and 10.00. Characteristic parameters of RLC (rα, I_k_, rETR_max_) were derived from computational curve-fitting using SigmaPlot12.3, following previously published methods ([29,30]; electronic supplementary material, Methods).

### 2.5. Photosynthesis, respiration and calcification

Net coral photosynthesis in the light and coral respiration in the dark was derived from rates of oxygen production/consumption taken during daytime in sealed, stir bar-agitated, temperature-controlled metabolic chambers (250–270 ml). Rates were determined for each fragment over one hour in the light (saturating light level of approximately 160 µmol m^−2^ s^−1^), followed by one hour in the dark under their respective treatment conditions. Oxygen saturation was monitored with an optode connected to an OXY-4 mini (Precision Sensing GmbH, Regensburg, Germany) and used to calculate the linear slopes of change in relative oxygen saturation after blank correction and accounting for the effects of temperature and salinity on oxygen saturation (electronic supplementary material, Methods). Note that only the steepest linear part of the curve was used, i.e. dark respiration values represent dark-acclimated dark respiration based on the last approximately 40 min of the incubation (cf. electronic supplementary material, table S4). Gross photosynthetic rates were derived from the equation: net photosynthesis (light) = gross photosynthesis (light) − respiration (dark), assuming a negligible difference between coral respiration in the light and dark. Productivity was expressed as daily productivity (P_gross_ : R ratio with 14 h : 24 h) and host O_2_ demand in the light was calculated as the direct R : P_gross_ ratio. Calcification rate was determined from the change in total alkalinity in water samples taken at the beginning and end of all respirometry measurements. Samples were titrated by a titrosampler (Compact Titrosampler, Metrohm AG, Herisau, Switzerland) and calcification rates determined accordingly [31].

Values for photosynthesis, respiration and calcification were normalized to relevant variables such as coral surface area, protein biomass, symbiont density or chlorophyll content.

### 2.6. Fragment processing and assessment of bleaching proxies

Coral fragments were brushed with a waterpik, using cold lysis buffer (50 mM KH_2_PO_4_/K_2_HPO_4_, 0.1 mM EDTA, 10% [v/v] glycerol, pH 7.0, 4°C), and the tissue briefly homogenized with a handheld homogenizer (30 s; DIAX 100, Heidolph Instruments, Schwabach, Germany). Symbionts of a 500 µl aliquot were pelleted (2000 × *g*, 5 min, 4°C) for later chlorophyll (chl) extraction. Another 500 µl aliquot was fixed in 4% formaldehyde for cell density determination. The remaining slurry was centrifuged (2000 × *g*, 5 min, 4°C) and the obtained symbiont pellet flash-frozen and stored at −80°C. The remaining host tissue was centrifuged (16 000 × *g*, 5 min, 4°C) and aliquots for the various biochemical measurements snap-frozen. For biochemical analysis of symbionts the pellet was washed three times in lysis buffer (1500 × *g*, 5 min, 4°C) and lysed in 600 µl of lysis buffer using a bead mill (Mini beadbeater, setting 1 (10 s); Glen Mills Inc., Clifton, NJ, USA,) with approximately 200 mg of glass beads (425–600 µm size). The resulting homogenate was centrifuged (16 000 × *g*, 5 min, 4°C), aliquoted and frozen at −80°C.

Brushed coral skeletons were dried and their surface area determined by wax dipping [32]. Symbiont chl was extracted in 1 ml of 90% acetone in the dark (24 h, 4°C). Samples were centrifuged (5000 × *g*, 4°C) and the concentration of chl *a* and *c_2_* in the supernatant spectrophotometrically determined [33]. Symbiont density was determined via haemocytometer counts (*N* = 3 per sample with s.d./mean <15%) and normalized to the coral surface area.

### 2.7. Biochemical analysis

The total carbohydrate content of host and symbiont tissue was assessed using an adapted protocol of the sulphuric acid–phenol method for microtitre plates ([34]; electronic supplementary material, Methods). All carbohydrate values are expressed using glucose as standard. The total soluble protein content of host and symbiont was determined with the improved Bradford protocol, using bovine serum albumin as the protein standard [35]. Measurements of the enzymatic antioxidants superoxide dismutase (SOD) and catalase (CAT in host) or catalase peroxidase (KatG in symbiont) were performed as previously described ([36]; electronic supplementary material, Methods).

### 2.8. Nanoscale secondary ion mass spectrometry experiment

Three of the nine (A, G, I) randomly selected *Stylophora pistillata* replicates from the ambient and elevated temperature treatments were used in an isotopic dual labelling experiment to assess the isolated effect of temperature stress on symbiont carbon and nitrogen fixation and translocation to the coral host. Experiments were conducted in 30 l aquaria with internal flow in which the seawater was isotopically labelled by adding 2 mM NaH^13^CO_3_ (98 atom %) and 3 µM K^15^NO_3_ (98 atom %). Coral nubbins from both temperature treatments were exposed to these conditions for a total of 6 h (10.00 to 16.00 local time) with a replacement of water every two hours to minimize the increase in water temperature due to high ambient air temperatures.

At the end of the isotopic pulse, the apical tip of the branch was removed and a 1 cm coral piece clipped off and immersed in fixative (2.5% [v/v] glutaraldehyde, 0.5% [v/v] formaldehyde in 0.22 µm filtered 0.1 M phosphate buffer with 0.6 M sucrose, pH 7.4–7.6) for 2 h at room temperature, followed by 22 h at 4°C. Pieces were subsequently washed and completely decalcified in 0.5 M EDTA. Fixed tissue samples were dissected to obtain connective coenosarc tissue and post-fixed in 1% [v/v] osmium tetroxide in water for 1 h. Post-fixed samples were dehydrated, embedded in Spur resin blocks and microtomed following published protocols ([37]; electronic supplementary material, Methods). Transmission electron microscopy (TEM) was carried out at 80 kV with a Philips CM 100 transmission electron microscope at the Electron Microscopy Facility (EMF) at the University of Lausanne (Switzerland).

Based on TEM imaging, areas of interest were analysed for ^13^C- and ^15^N-enrichment using the NanoSIMS 50 l ion microprobe at the Laboratory for Biological Geochemistry at EPFL, Lausanne, Switzerland. Ultrathin sections (70 nm) on TEM grids and corresponding semi-thin (500 nm) sections on glass coverslips were gold-coated and bombarded with a 16 keV primary Cs^+^ ion beam. Raster scans of the area of interest (6 layers) were performed with a primary beam spot size of around 150 nm. Image resolution was set to 256 × 256 pixels with a beam dwell time of 5 ms per pixel. Image size was set to 40 × 40 µm, which provided a complete cross section of the epidermis and oral gastrodermis with sufficient resolution for subcellular features to be clearly resolved. Approximately 10–15 images were obtained per sample in order to cover enough area to image 50–60 symbiont cells and their surrounding host tissue. In case multiple sections from the same sample block were required to obtain enough cells, they were taken at least 20 µm apart to avoid cutting and imaging another layer of the same symbiont cells. The secondary ions ^12^C_2_^−^, ^13^C^12^C^−^, ^12^C^14^N^−^ and ^12^C^15^N^−^ were simultaneously counted at a mass resolution of about 10 000 (enough to resolve all potential interferences) in electron multipliers.

NanoSIMS data were processed using the L'IMAGE software (created by Dr Larry Nittler, Carnegie Institution of Washington). Maps of ^13^C/^12^C and ^15^N/^14^N enrichment were derived from drift-corrected image ratios of ^13^C^12^C^−^ and ^12^C_2_^−^, and ^12^C^15^N^−^ and ^12^C^14^N^−^, respectively. Quantification of isotopic abundance in different compartments was achieved by drawing regions of interest (ROIs) on ratio images. The following ROIs were defined and manually drawn based on contour outlines visible on the ^12^C^14^N^−^ image and/or both ratio images: Symbiont, host gastrodermis (excluding symbionts), host epidermis and host lipid droplets. Isotopic enrichments were converted to atom percent excess (APE) [38], which represents the fraction of replaced C or N in a steady state, i.e. assuming a negligible increase in total biomass within the pulse period (electronic supplementary material, Methods).

### 2.9. Statistical analysis

The effects of temperature and pH on the physiological parameters were tested as part of a factorial design with two levels for each factor. All treatments were replicated in three separate tanks with three different coral colonies per tank. Data were analysed with a linear mixed model with a restricted maximum-likelihood approach (REML), using temperature, pH and replicate group as fixed factors, and replicate as a random factor. While the factors temperature and pH were crossed to assess their combined effect, replicate tank (I, II, III) was only used as a main factor in the model in order to test whether there was a difference due to the replicate tank that was consistent across all treatments. Replicate (nested within replicate tank) was included as a random factor to assess how much of the variance in the experiment was attributed to the randomly selected colony replicates (variance components analysis). When high interaction terms were not significant, the full factorial model was reduced to a minimal adequate model. All dependent variables were tested for normal distribution using the Shapiro–Wilk test and when necessary were transformed to achieve normality. Tukey's HSD test was applied as a *post hoc* test where appropriate. All statistical analyses were performed using the software JMP v. 11.2.1 (SAS Institute, Cary, NC, USA).

Effects of temperature on carbon and nitrogen assimilation (NanoSIMS data) were tested in paired *t*-tests for each class of ROI. Data were checked for normality and homoscedasticity using the Shapiro–Wilk test and Levene's test, respectively, and transformed if necessary.

## 3. Results

**Figure 3.**
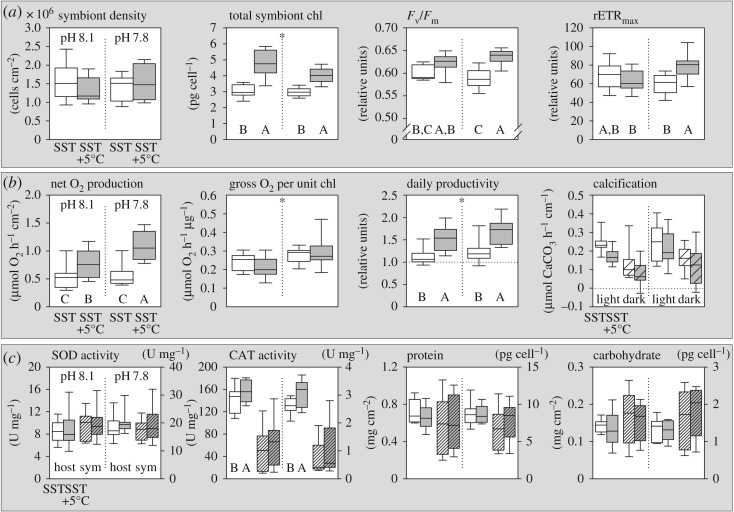
Physiological response of *Stylophora pistillata* to elevated temperature and reduced pH. All graphs show responses to ambient (white) versus high temperatures (grey) under ambient (left side) and reduced pH conditions (right side), respectively. (*a*) Coral symbiont characteristics and photochemistry. (*b*) Coral productivity and calcification in the light (clear box) and dark (textured box). (*c*) Host (clear boxes, left axis) and symbiont (textured boxes, right axis) enzymatic antioxidant activity (normalized to protein) and total soluble protein and carbohydrate content. Letters indicate significant *post hoc* differences between boxplots with asterisks indicating overall pH effects (electronic supplementary material, table S1; *N* = 9).

After six weeks of exposure to elevated temperature and a pH of 7.8, none of the colonies showed visual signs of bleaching. Indeed, symbiont densities were not significantly affected by any of the treatments (figure 3*a*; electronic supplementary material, table S1) and symbiont total chlorophyll concentration significantly increased by 44% in the combined stress scenario, predominantly driven by temperature (+45%), compared to pH (−9%) (figure 3*a*; electronic supplementary material, table S1). Variables related to electron flow in photosystem II showed a synergistic effect of pH and elevated temperature: rapid light curves indicated significantly higher maximum quantum yields of photosystem II (*F*_v_/*F*_m_; +6%) with increased maximal relative electron transport rates (rETR_max_; +19%) (figure 3*a*). These changes in pigmentation and photochemistry corresponded to an increased light demand to saturate photosynthesis (I_k_; +16%) and decreased chl *a*/chl *c_2_* ratio (−10%) at elevated temperatures (electronic supplementary material, figure S4*a–c*).

As a consequence of the increase in the light reaction of photosynthesis, gross (P_gross_) and net oxygen production (P_net_) per surface area were improved substantially by exposure to elevated temperatures (+83%) and effectively doubled (+129%) in the combined treatment (figure 3*b*). Holobiont respiration showed minor, non-significant changes (+7%) under these conditions (electronic supplementary material, figure S4*e* and table S1). Notably, reduced pH had an additive positive effect on net oxygen production only at elevated temperature and not under ambient conditions. Reduced pH also significantly enhanced the efficiency of gross oxygen generation per unit of chlorophyll, independent of temperature (figure 3*b*). As a result of this profound shift in symbiont photosynthesis with temperature and minor changes in holobiont respiration, the amount of respired O_2_ relative to the total production (Host O_2_ demand; R : P_gross_) significantly declined by almost a third (electronic supplementary material, figure S4*f*). Scaling the metabolic oxygen data to a daily budget (P_gross_ : R ratio for 14 h : 24 h; figure 3*b*) indicates a daily productivity for the coral holobiont of 1.14 ± 0.22 (mean ± s.d., *N* = 9) under ambient conditions. This ratio significantly increased to 1.72 ± 0.33 under the combined pH/temp treatment (figure 3*b*). Again, this was driven by a strong positive effect of temperature (+39%), although reduced pH also enhanced this ratio (+12%).

The increase in holobiont productivity did not translate to significant changes in coral calcification or energetic proxies such as carbohydrate and protein content of host and symbiont. While coral calcification tended to decline slightly under elevated temperatures, neither light nor dark calcification was significantly affected across treatments (figure 3*b*; electronic supplementary material, table S1). Protein and carbohydrate content in symbionts and host tissue showed no significant changes in any treatments after two months of exposure (figure 3*c*; electronic supplementary material, table S1). Of the antioxidant markers, only catalase activity in the host tissue was elevated in response to higher temperatures (CAT; +14%; figure 3*c*), but this was not accompanied by higher host superoxide dismutase (SOD). Activity of these two antioxidant enzymes did not significantly change in the symbiont population in any treatment (figure 3*c*).

### 3.1. The effects of elevated temperature on carbon and nitrogen utilization

**Figure 4.**
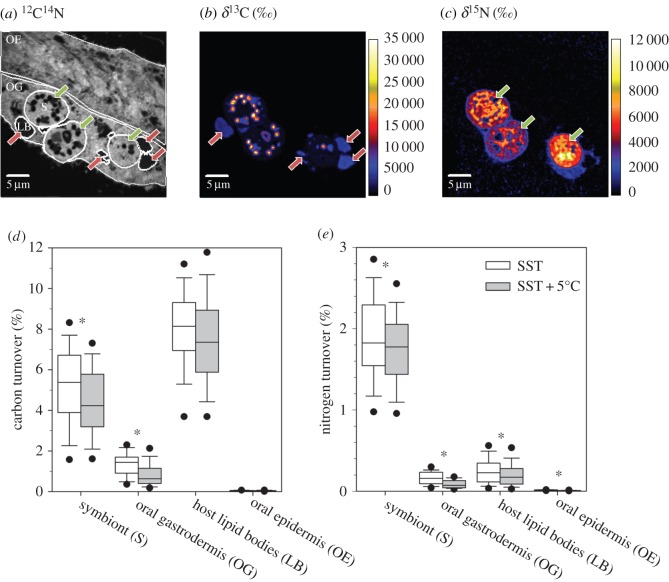
Autotrophic uptake and translocation of C and N at the (sub-)cellular level in the coral symbiosis. (*a*) Typical NanoSIMS ^12^C^14^N^−^ image showing ultrastructure of the oral epidermis (OE) and gastrodermis (OG); the latter contains symbionts (S) and lipid bodies (LBs). (*b,c*) ^13^C and ^15^N enrichment from symbiont fixation of [^13^C]-bicarbonate and [^15^N]-nitrate over 6 h in the light. (*d,e*) Fractions of replaced C and N, respectively, under ambient (white) and elevated (grey) temperature at ambient pH (asterisks indicate significant differences; electronic supplementary material, table S3). Boxplots depict all values from three replicates (electronic supplementary material, table S2).

Temperature was observed to be the main driver for changes in photosynthesis and productivity of the coral holobiont. Therefore, after two months of exposure, we performed a 6 h incubation with [^13^C]-bicarbonate and [^15^N]-nitrate in the light at ambient and elevated temperature (cf. electronic supplementary material, Methods) to investigate rates and allocation of photosynthetically fixed nutrients. Combined with transmission electron microscopy (data not shown) and NanoSIMS isotopic imaging [39], this provided detailed information about the anabolic fate of photosynthetically assimilated nutrients and their turnover in different subcellular compartments under ambient and elevated temperature conditions in the oral tissue (figure 4*a–c*). Symbionts primarily store photosynthetically fixed C as starch and lipids, which showed the highest ^13^C-enrichments (figure 4*b*). Assuming steady-state conditions, 5.5 ± 0.4% of the total symbiont structural C was replaced over the 6-hour incubation period under ambient temperatures, whereas 8.0 ± 0.2% of C in host lipids was replaced in the same time period (figure 4*d*). Almost all translocated C (97–100%) and N (91–95%) was detected in the oral gastrodermis, rather than the epidermis (figure 4*d,e*; electronic supplementary material, table S2).

NanoSIMS imaging of the polyp-connecting coenosarc tissue showed reduced symbiont C and N fixation and subsequent host assimilation under elevated temperatures (figure 4*d,e*; electronic supplementary material, tables S2 and S3), although strong variability was observed at this cellular level between three replicates (electronic supplementary material, table S3). In the following we therefore indicate in parenthesis the full range of observed responses. At elevated temperatures, coral symbionts displayed an average change in the amount of fixed C and N of −17% (+1 to −31%) and −6% (+26 to −30%), respectively (figure 4*d,e*). Likewise, both the host gastrodermis (−35%) and epidermis (−36%) assimilated less translocated N (−14 to −78% and −14 to −52%, respectively), whereas reduced C assimilation (−31%) was only detected for the gastrodermis (−1 to −65%).

The major stores for translocated C were lipid bodies (LBs) in the host gastrodermis with an average size of 2.6 ± 1.3 µm (*N* = 282). Allocation of photosynthetic C to these reservoirs tended to decline at higher temperature, but this was not statistically significant (−9 ± 23%; *p* = 0.0503). While translocated N was not found in specific hotspots in the host tissue, it tended to be more concentrated in the vicinity of the symbiosomes containing the symbionts (figure 4*c*). Interestingly, the fraction of replaced N in host LBs was comparable to that in the gastrodermis as a whole (figure 4*e*; electronic supplementary material, table S2) and while elevated temperature significantly reduced the N turnover in these structures by 21% (−5 to −41%), its impact was smaller than in the overall gastrodermis (figure 4*e*).

## 4. Discussion

*Stylophora pistillata* from the GoA showed extreme resistance to the combined stress of elevated temperature and reduced pH over the experimental monitoring period that exposed the coral to an extended period (six weeks) with up to 2°C above its long-term summer maximum of 27.67 ± 0.75°C (figure 2). In nature, this coral currently does usually not spend more than 2 days per year above our initial treatment temperature of 28°C in the shallow waters of the GoA (exception of 2007 and 2012 with 10 days), where the annual mean SST is 24.1 ± 2.0°C [22]. The observations presented here demonstrate the high resistance of *S. pistillata* corals from the GoA to simulated future ocean conditions. Simulated ocean warming and reduced pH had no major negative impact on the stability of the symbiosis, confirming previous suggestions of high physiological plasticity and acclimation potential of corals from the Northern Red Sea [20,40]. While the focus of our study was to test a realistic scenario for a common reef-building coral in the second half of this century, previous experiments have shown the robustness of other GoA corals to even higher temperature excursions [20]. Our study demonstrates that an elevation of the SST increases the photosynthetic performance of the dinoflagellate population in *S. pistillata,* which has been exclusively identified as predominantly *Symbiodinium* ITS2 type A1 in the shallow water specimen used here [41]. The pigment, fluorometric and oxygen data indicate that enhanced light harvesting through primary and accessory chlorophylls allows increased electron flow and oxygen release in photosystem II. Because symbiont density as well as host respiration was not affected in any of the treatments, enhanced photosynthesis in the symbionts can be identified as the main driver of the observed increase in coral productivity. These changes in symbiont photosynthesis translated directly into an increased oxygen efflux from the colony surface. This effect was consistent across nine coral replicates, despite variability in their resident symbiont density (ranging from 0.9 to 2.6 × 10^6^ cells cm^−2^). A greater oxygen efflux from the colony surface might improve local water oxygenation during the day and therefore locally attenuate the negative effects of decreased oxygen solubility in warmer water (a drop of −9% between 23°C and 28°C), benefiting aerobic biological processes that are accelerated under elevated temperature, such as microbial activity and fish respiration [42,43]. From a cellular perspective, higher tissue oxygenation with elevated *p*O_2_ tends to promote the generation of reactive oxygen species [44], especially in photosymbiotic animals [45], but we found no indication of upregulation of two major enzymatic antioxidants in either partner. We interpret the increase in host catalase, independent of SOD activity and in the absence of bleaching, as an indication for higher metabolic activity at elevated temperature.

Under ambient conditions, the daily productivity of *Stylophora pistillata*, based on oxygen metabolism, indicated a balanced metabolic budget, similar to the estimated total productivity of a coral reef community of around one [46]. The considerable increase in P_gross_ : R ratios under elevated temperature and reduced pH was, however, not reflected in changes in symbiont or host protein biomass or carbohydrate content that one would expect to accompany improved photosynthesis. An increase in skeletal extension rate could have partly masked an increase in colony tissue growth, since it is normalized to surface, but no significant change in overall calcification was detected. It thus seems conceivable that the coral host experienced a shift in the relative activity of catabolic versus anabolic processes with productivity increases primarily invested into energy metabolism rather than tissue/skeletal growth.

The NanoSIMS data provided evidence that elevated temperature causes subtle declines in C and N acquisition in the coral tissue that were, however, not reflected in the biomass proxies within the experimental observation period. Considering the increased oxygen productivity and the absence of photoinhibition or elevated SOD activity (figure 3*a–c*), we interpret the NanoSIMS results as changes in the utilization efficiency of CO_2_ by Rubisco and a decoupling between the light and dark reactions of photosynthesis at elevated temperatures. The specific constellation of increased O_2_ production and decreased C-fixation points towards photorespiratory mechanisms, which might also be stimulated by the low ability of the dinoflagellate type-II Rubisco to discriminate between O_2_ and CO_2_ [47]. In this regard photorespiration acts as an overflow mechanism, because demand for ATP and NADPH is much higher for Rubisco oxygenation than carboxylation [48]. This mechanism would allow the coral symbiont to maintain high electron transport rates and light utilization without risking severe photoinhibition [49–51]. The lower N-fixation and translocation at elevated temperature is also consistent with a lower symbiont C fixation and illustrates the tight co-regulation of C- and N-pathways in photosynthetic organisms; i.e. the use of C-skeletons for amino acid metabolism and structural protein synthesis [52]. Given the complex biochemistry of photorespiration and the involvement of three different organelles, intermediates such as glycolate and glycine will find their way into the host tissue as part of a suite of translocated photosynthates. Such a leakage of photorespiratory intermediates would contribute to a reduced activity of the Calvin–Benson cycle in the symbionts and could explain why the host showed no change in the proportion of symbiont-derived carbon in host LBs at higher temperatures despite lower initial symbiont fixation rates and lower overall C-incorporation in the gastrodermis. Carbon turnover in host LBs was higher than in the overall symbiont cell (figure 4*d*), confirming that these structures are the major transient C stores in the host tissue [37]. While comparable levels of N-labelling in host LBs and gastrodermis indicated the synthesis of N-containing lipids and/or LB-associated proteins within the 6 h pulse, we cannot conclude with certainty that the reduced N-labelling of LBs at elevated temperature simply reflects the lower fixation and translocation from the symbiont. It could also reflect changes in LB composition, i.e. in the abundance and activity of LB-associated proteins known to be associated with the cellular pathways of energy metabolism, cytoskeleton dynamics and intracellular trafficking [53]. In general, it should be noted that conventional sample preparation for NanoSIMS involves a loss of most soluble compounds and restricts observations mainly to the anabolic part of metabolism. Thus, changes in C-labelling can also be indicative of higher catabolic activity and enhanced shuttling of intermediates through the TCA cycle as part of cata- and anapleurotic reactions, which would release ^13^CO_2_ from the system.

### 4.1. Selected for heat tolerance, but currently living at suboptimal temperatures

Our experiment showed that *Stylophora pistillata* from the GoA is resistant to summer conditions to be expected for the second half of the century. The positive effect of elevated temperature on key physiological parameters suggests that these corals are currently living at suboptimal temperatures in the Northern Red Sea in comparison to specimens in the Central and Southern Red Sea, where the annual and summer mean SST are much higher. Thus, under continuous warming, it is likely that *S. pistillata* in the GoA will actually continue to show improved performance until its thermal threshold is approached. Our findings strongly underpin previous suggestions on the temperature resistance of corals in the GoA as a result of their evolutionary history [20,54,55]. Colonized by corals that have undergone such evolutionary selection for high thermal tolerance in the warm waters of the Central and Southern Red Sea, the highly diverse coral reef in the GoA represents a unique source: these corals live in the Northern Red Sea at suboptimal temperatures, much below their thermal threshold, and might provide a genetic reservoir in the future, capable of restocking decimated coral reefs in other parts of the Red Sea [20,40].

Despite these positive physiological data, further work on the effects of elevated temperature and reduced pH is required to understand the ecological implications with regard to recruitment success, growth rates and competitive performance. Long-term sublethal effects are equally important to consider. For example, reduced calcification in the absence of obvious bleaching is still a major problem for overall reef growth, as shown for *Diploastrea heliopora* in the Central Red Sea [56]. Red Sea *S. pistillata* calcification appears robust against the combination of elevated temperature and reduced pH in our experiment and other work on a Red Sea specimen has demonstrated that it can maintain calcification rates and skeletal integrity at a pH of 7.8 under ambient temperature for even longer exposure periods (more than 1 year) [57].

Loya [26] suggested that *S. pistillata* is an r-strategist coral, given its rapid growth rate, early reproductive age and tendency to colonize unpredictable habitats. Another support of the species being a typical r-strategist stems from a massive die-off of *S. pistillata* following local environmental disturbances such as an anomalously low tide. The present study demonstrates physiological resistance of this species to global environmental change in the marine environment (ocean warming and acidification). While the latter gives hope of survival under ‘business-as-usual’ climate change scenarios, it has to be considered that local disturbances, including pollution, eutrophication and physical damage may lower the capacity of *S. pistillata* to withstand global future environmental conditions.

Considering the rapid decline of coral reefs worldwide, our study emphasizes the urgent need to reduce local stressors in the GoA region. Physical damage, pollution and eutrophication amplify the impact of global stressors. The semi-enclosed GoA currently harbours extensive fringing reefs and sea grass meadows, but it is also subject to increasing pressure from industrial and urban developments. Previous case studies have demonstrated the devastating impact that pollution and wastewater discharge can have on the local coral population in the GoA [58,59]. It is therefore imperative to enact an effective, trans-national environmental protection and management treaty in the wider Red Sea region [60]. These corals might stand a chance of surviving the global trends of ocean warming and acidification, but only if decisive actions are taken now to reduce the impact of local threats.

## Supplementary Material

Extended Materials & Methods

## Supplementary Material

ESM material titles and captions

## Supplementary Material

Figure S1. Experimental setup and long-term warming trends

## Supplementary Material

Figure S2. Schematic of the experimental layout in the IUI's Red Sea Simulator aquaria array

## Supplementary Material

Figure S3. Monitoring data of experimental setup

## Supplementary Material

Figure S4. Physiological variables of Stylophora pistillata under elevated temperature and reduced pH

## Supplementary Material

Table S1. Statistical output of mixed model analysis with indicated effect size for significant factors

## Supplementary Material

Table S2. Summary of replicate-specific turnover values for carbon and nitrogen from NanoSIMS image analysis

## Supplementary Material

Table S3. Statistical ouput for NanoSIMS data

## Supplementary Material

Table S4. Alkalinity and oxygen saturation in respiration chambers

## Supplementary Material

Table S5. Physiological raw data

## Supplementary Material

Table S6. Two-way ANOVA outputs testing for a consistent effect of mixing tank for the pH 7.8 treatments for all main physiological variables presented in Fig. 3

## Data accessibility

The supporting data are provided in electronic supplementary material, table S2 (NanoSIMS data) and table S5 (Physiological data).
