# Supplementary material for: Common reef-building coral in the Northern Red Sea resistant to elevated temperature and acidification
Source: R Soc Open Sci. 2017 May 17;4(5):170038. doi: 10.1098/rsos.170038 (PMC5451809; doi:10.1098/rsos.170038)
Supplement: Extended Materials & Methods [file rsos170038supp1.docx]

## Extended Materials & Methods

*Experimental settings*

Water flowing to the experimental aquaria went through 4 large reservoir tanks (2000 L) that allowed modification of pH (NBS scale) by bubbling with CO_2_ (Fig S2). Outflowing water from the reservoir to the aquaria was monitored for temperature and pH with an automatic feedback and control of the pH level in the reservoir tank and the set temperature in individual aquaria. Temperature in heated aquaria (the experimental units) was controlled individually through a heater-cooler closed system and a titanium heat exchanger setting the temperature at a pre-defined delta to the inflowing ambient water temperature. In addition, the complete aquaria system was monitored by a computerized robot, moving along the aquaria lines and measuring temperature (typical resistance thermometer PT100), dissolved oxygen saturation (VisiFerm DO ARC 120, Hamilton, Switzerland) and pH levels (POLILYTE PLUS ARC 120, Hamilton, Switzerland) on an hourly basis (Fig S1c; Fig S3). Carbonate chemistry was monitored by taking three independent measurements of water samples from three consecutive days for the time points 1 (27-29/04/14), 2 (18-20/05/14), and 3 (05-07/06/16). Each daily value is based on three water samples taken in the morning, noon, and afternoon (treated as technical replicates per day). Carbonate chemistry was calculated using the excel macro CO2Sys V.2.1. The thermodynamic carbonate dissociation constants for activity scales (K1 = 5.845±0.008 and K2 = 8.945±0.013) were attained from [[1](#_ENREF_1)] and the refit from [[2](#_ENREF_2)]. The input parameters for pressure (10 dbars), total P (0.027 µmol kg^-1^ SW) and total Si (0.69 µmol kg^-1^ SW) were obtained from the National Monitoring Program (NMP) at the Gulf of Eilat [[3](#_ENREF_3)]. Settings for KHSO_4_ and [B]_T_ were chosen after [[4](#_ENREF_4)] and [[5](#_ENREF_5)], respectively. Reported pH values refer to NBS scale (pH_NBS_) unless explicitly outlined. Where pH is expressed on a total scale (pH_T_), 0.11 pH units have been substracted from pH_NBS_ [[6](#_ENREF_6)].

*PAM fluorometry*

Measurements of minimal (F_o_) and maximal (F_m_’) fluorescence at each PAR level, with F_m_’-F_o_ = F_v_ (variable fluorescence) were used to calculate the electron transport rate (ETR = F_v_/F_m_’ x PAR x ETR-factor), where the ETR-factor is the product of the amount of total PAR absorbed and its allocation to photosystem II (PSII) [[7](#_ENREF_7), [8](#_ENREF_8)]. Since the ETR-factor was not determined for each fragment, it was set to 1. Thus, all presented ETR- and alpha-values are relative values (rETR; rα). Maximum quantum yield of PSII (F_v_/F_m_) was derived from the first yield measurement of the RLC.

*Photosynthesis, respiration*

Net coral photosynthesis in the light and coral respiration in the dark was derived from rates of oxygen production/consumption in sealed acrylic-glass metabolic chambers (250-270 mL) with a double wall and temperature-adjusted water flow, serving as a water jacket (adjusted according to treatment temperatures). Seawater was continuously mixed using a magnetic stirrer to ensure dissolved oxygen was homogenous. Incubation times were on average 1-1.5h in order to have sufficient changes in TA while at the same time limiting changes to carbonate chemistry and oxygen saturation (Table S4). Net oxygen production in the light and respiration in the dark per fragment [∆O_2_ as mg O_2_ min^-1^] were calculated from the linear slope of changes in relative oxygen saturation after blank correction (∆O_2_ as % min^-1^), while accounting for the effects of temperature and salinity on oxygen saturation (DO) according to the following equation:

∆O_2_ [mg min^-1^] = ∆O_2_ [% min^-1^] x DO_23°C/28°C_ [mg L^-1^] x (V_chamber_ [L] – V_coral_ [L])

Oxygen saturation levels were set as 6.809 (23°C) and 6.263 mg L^-1^ (28°C) for a salinity of 40 [[9](#_ENREF_9)]. Volumes of live coral fragments (V_coral_) were determined to accurately calculate the used incubation volume in the respiration chamber. Coral volumes were determined by weighing the seawater displacement of suspended, submerged fragments, following Archimedes' principle and assuming an approximate density of 1.028 g cm^-3^ for seawater in the Red Sea. Oxygen data were converted into micromole (using 31.9988 g mol^-1^) and normalized to either surface area or total chlorophyll content.

*Biochemical analysis*

Total carbohydrate content of host and symbiont tissue was assessed using an adapted protocol of the sulphuric acid-phenol method for microtiter plates[[10](#_ENREF_10)]. 50 µL of sample or glucose standard (0-1.6 nmol µL^-1^) in lysis buffer was rapidly mixed with 150 µL of concentrated sulfuric acid and 30 µL of 5% [v/v] phenol in water in a 1.5 mL tube. Tubes were incubated at 90°C for 5 min in a water bath, cooled to room temperature, and the absorbance of 3x200 µL measured at 493 nm in a spectrophotometer. All carbohydrate values are expressed as glucose equivalents.

Total soluble protein content of host and symbiont was determined with the improved Bradford protocol, using bovine serum albumin as protein standard [[11](#_ENREF_11)].

Measurements of the enzymatic antioxidants superoxide dismutase (SOD) and catalase (CAT in host) or catalase peroxidase (KatG in symbiont) were performed as previously described [[12](#_ENREF_12)]. Briefly, CAT and KatG activity were determined spectrophotometrically in quartz cuvettes at 240 nm using 100 μL lysate (1:10 diluted for host samples) in a total reaction volume of 700 μL, containing potassium phosphate buffer (50 mM, pH 7.0), EDTA (0.1 mM), and hydrogen peroxide (14 mM). Linear kinetics were monitored for 3 min at 25°C and activities calculated using an extinction coefﬁcient of 43.6 M^-1^ cm^-1^ (ref. [[13](#_ENREF_13)]). SOD assays were performed using the riboﬂavin/nitroblue tetrazolium (RF/NBT) assay in a microtiter plate format [[14](#_ENREF_14), [15](#_ENREF_15)]. 20 µL of sample lysate or SOD standards (0.5-500 U/mL) were measured in a ﬁnal reaction mixture of 300 μL potassium phosphate buffer (50 mM, pH 7.8) containing EDTA (0.1 mM), riboﬂavin (1.3 μM), L-methionine (10 mM), NBT (57 μM), and Triton X-100 (0.025% [v/v]). Absorbance was read at 560 nm both immediately and after 5 minutes incubation under a homogeneous light ﬁeld (200 μmol quanta m^-2^ s^-1^) at 25°C using a microtiter plate reader. Standards and samples were measured using the same reaction mixture and a sigmoidal 5-parameter semi-logarithmic standard curve (24 equally spaced standard levels) was used to determine SOD activity of samples. One unit of SOD activity inhibited the NBT reduction by 50%.

*NanoSIMS sample preparation*

Post-fixed samples were processed following underwent a series of ethanol dehydration (3 changes of 10 min in 50%, 70%, 90%, 100%), mixed Spurr resin-ethanol infiltration (3 x 24h steps of 1+2, 1+1, and 3+1 parts resin:ethanol), and pure Spurr infiltration (3 x 12h) and embedment. Resin blocks were cut with a Diatome 45° diamond knife to obtain semi-thin sections (500 nm) for direct NanoSIMS analysis and ultrathin sections (70 nm) for correlative TEM and NanoSIMS analysis. Semi-thin sections were mounted on round glass coverslips without any staining. Ultrathin sections were mounted on Formvar carbon-coated alphanumeric grids, counterstained with 4% uranyl acetate (10 min) and Reynold’s lead citrate solution (10-30 sec) for ultrastructural observation.

*NanoSIMS data analysis*

Quantification of isotopic abundance in different compartments was achieved by drawing regions of interest (ROIs) on ratio images, providing the respective isotope ratios. In order to be able to judge whether or not specific structures are enriched in our treatment samples, the same ROIs were also quantified in an independent unlabelled reference sample in order to obtain a measure of noise (which is size-dependent) for a given class of ROI. Samples were considered enriched if the mean value for each type of ROI was more than three standard deviations above the average obtained from similar ROIs in the unlabelled reference samples.

In order to obtain relative turnover numbers for carbon and nitrogen in different compartments, absolute isotope ratios were calculated by converting isotope ratios (R) of the sample and the reference to atom fraction (F), following the equation used for two stable isotopes [[16](#_ENREF_16)]:

$F_{sample}[\%]= \frac{R_{sample}}{1+R_{sample}}$ [1]

Atom fractions of ^13^C (*F*_SW_C_) and ^15^N (*F*_SW_N_) in the spiked seawater were calculated assuming standard atom fractions for carbon (*F*_PDB_: 0.01111) and nitrogen (*F*_nitrogen_: 0.00366) [[16](#_ENREF_16)] in the natural seawater and a concentration of 2.2 mM total dissolved inorganic carbon and 0.3 µM nitrate. Both equations express the atom fraction in 1L of seawater. For the spike, atom fractions were 0.98 for both ^13^C and ^15^N (Sigma Aldrich).

$$F_{SW_{C}}=\frac{{(n}_{SW DIC} x F_{PDB})+{(n}_{SPIKE DIC} x F_{SPIKE DIC})}{n_{SW DIC}+n_{SPIKE DIC}}$$

$=\frac{\left( 2.2 x 0.0111 \right)+(2.0 x 0.98)}{2.2+2.0}=0.4725$ [2]

$$F_{SW_{N}}=\frac{{(n}_{SW nitrate} x F_{nitrogen})+{(n}_{SPIKE nitrate} x F_{SPIKE nitrate})}{n_{DIC nitrate}+n_{SPIKE nitrate}}$$

$=\frac{\left( 0.3 x 0.00366 \right)+(3.0 x 0.98)}{0.3+3.0}=0.8912$ [3]

The fraction of replaced C or N in each compartment was expressed as atom percent excess (APE) [[16](#_ENREF_16)], using the element-specific *F*_SW_ values given in equation [2] and [3] according to:

$f=\frac{F_{sample}-F_{reference}}{F_{SW\_C/SW\_N}-F_{PDB/nitrogen}}x 100$ [4]

## References

1 Mehrbach, C., Culberson, C. H., Hawley, J. E., Pytkowicz, R. M. 1973 Measurement of the apparent dissociation constants of carbonic acid in seawater at atmospheric pressure. *Limnol Oceanogr*. **18**, 897-907.

2 Dickson, A., Millero, F. J. 1987 A comparison of the equilibrium constants for the dissociation of carbonic acid in seawater media. *Deep Sea Research Part A. Oceanographic Research Papers*. **34**, 1733-1743.

3 NMP. The Israel National Monitoring Program (NMP) at the Gulf of Eilat. Viewed 15/11/16. <http://www.iui-eilat.ac.il/Research/NMPmeteodata.aspx>. 2016.

4 Dickson, A. G. 1990 Standard potential of the reaction: AgCl (s)+ 1/2 H_2_ (g)= Ag (s)+ HCl (aq), and and the standard acidity constant of the ion HSO_4_^-^ in synthetic sea water from 273.15 to 318.15 K. *The Journal of Chemical Thermodynamics*. **22**, 113-127.

5 Lee, K., Kim, T.-W., Byrne, R. H., Millero, F. J., Feely, R. A., Liu, Y.-M. 2010 The universal ratio of boron to chlorinity for the North Pacific and North Atlantic oceans. *Geochim Cosmochim Acta*. **74**, 1801-1811.

6 Zeebe, R. E., Wolf-Gladrow, D. 2001 *CO_2_ in seawater: Equilibrium, kinetics, isotopes*. Amsterdam: Elsevier Science, B.V.

7 Genty, B., Briantais, J.-M., Baker, N. R. 1989 The relationship between the quantum yield of photosynthetic electron transport and quenching of chlorophyll fluorescence. *Biochim Biophys Acta*. **990**, 87-92.

8 Schreiber, U., Bilger, W., Neubauer, C. 1995 Chlorophyll fluorescence as a nonintrusive indicator for rapid assessment of *in vivo* photosynthesis. In *Ecophysiology of photosynthesis*. (ed.^eds. E.-D. Schulze, M. M. Caldwell), pp. 49-70. Berlin, Heidelberg: Springer.

9 Benson, B. B., Krause Jr., D. 1984 The concentration and isotopic fractionation of oxygen dissolved in freshwater and seawater in equilibrium with the atmosphere. *Limnol Oceanogr*. **29**, 620-632.

10 Masuko, T., Minami, A., Iwasaki, N., Majima, T., Nishimura, S.-I., Lee, Y. C. 2005 Carbohydrate analysis by a phenol–sulfuric acid method in microplate format. *Anal Biochem*. **339**, 69-72.

11 Zor, T., Selinger, Z. 1996 Linearization of the Bradford protein assay increases its sensitivity: theoretical and experimental studies. *Anal Biochem*. **236**, 302-308.

12 Krueger, T., Hawkins, T. D., Becker, S., Pontasch, S., Dove, S., Hoegh-Guldberg, O., Leggat, W., Fisher, P. L., Davy, S. K. 2015 Differential coral bleaching - Contrasting the activity and response of enzymatic antioxidants in symbiotic partners under thermal stress. *Comp Biochem Physiol A Mol Integr Physiol*. **190**, 15-25.

13 Beers, R., Sizer, I. W. 1952 A spectrophotometric method for measuring the breakdown of hydrogen peroxide by catalase. *J Biol Chem*. **195**, 133-140.

14 Beauchamp, C., Fridovich, I. 1971 Superoxide dismutase: Improved assays and an assay applicable to acrylamide gels. *Anal Biochem*. **44**, 276-287.

15 Fryer, M. J., Andrews, J. R., Oxborough, K., Blowers, D. A., Baker, N. R. 1998 Relationship between CO_2_ assimilation, photosynthetic electron transport, and active O_2_ metabolism in leaves of maize in the field during periods of low temperature. *Plant Physiol*. **116**, 571-580.

16 Slater, C., Preston, T., Weaver, L. T. 2001 Stable isotopes and the international system of units. *Rapid Commun. Mass Spectrom.* **15**, 1270-1273.
