# Supplementary material for: Common reef-building coral in the Northern Red Sea resistant to elevated temperature and acidification
Source: R Soc Open Sci. 2017 May 17;4(5):170038. doi: 10.1098/rsos.170038 (PMC5451809; doi:10.1098/rsos.170038)
Supplement: ESM material titles and captions [file rsos170038supp2.docx]

ESM 1

Title: Figure S1. Experimental setup and long-term warming trends.

Caption: (a) Typical fringing coral reefs in Eilat, Israel. (b) *Stylophora pistillata* fragments were acclimated and tested in the (c) Red Sea Simulator flow-through aquaria array. (d) Monthly long-term SST of the GoA, Eilat area (1988-2016). Linear regressions (black lines) with 95% confidence (dark grey shade) and prediction intervals (light grey shade) are outlined. Colouration indicates SST anomaly relative to the long-term average for each month. Data are derived from Fine et al. (2013) for 1988-2006 and from the National Monitoring Program at the Gulf of Eilat for 2007-2016. (e) Decadal warming rates for each month (mean±SE); all rates are significantly different from zero with p<0.0001 (Linear regression analysis).

ESM 2

Title: Figure S2. Schematic of the experimental layout in the IUI’s Red Sea Simulator aquaria array.

Caption: The whole system is a flow-through system with constant seawater influx, pH and temperature manipulation, and subsequent efflux (aquarium flow rate > 60L hour^-1^). Similar colours indicate aquaria supplied with the same water from their respective reservoir tanks (1-4). The setup allows heating of #109-128. For the experiment only aquaria #109-120 and #209-220 were available and replicate triplets (I-ABC, II-DEF, III-GHI) and treatments were randomly assigned within the heated and the control aquarium line. Aquaria-specific treatments are indicated for elevated temperature (°C) and/or reduced pH (pH).

ESM 3

Title: Figure S3. Monitoring data of experimental setup.

Caption: (a) Applied temperature profiles under ambient (grey) and elevated temperature (red; +5°C). (b) Applied seawater pH_NBS_ profiles at ambient pH 8.1 (circles) and reduced pH 7.8 (squares) for ambient and elevated temperature (grey vs. red). Values represent daily means across aquaria. Note that the slight offset of the measured pH values between both temperature treatments is probably the result of the pH shift due to temperature (since pH is adjusted in the main reservoir tank and does not account for later temperature manipulation in the aquaria; see Supplementary Methods). (c) Hourly daylight PAR levels for 30-day intervals (black: 10/04-08/05/14; light grey: 09/05-08/05/14; dark grey: 09/05-07/06/14; means±SD, N=30). (d) Carbonate chemistry of treatments over time. Values represent means±SD of three independent measurements from three consecutive days for time point 1 (27-29/04/14), 2 (18-20/05/14), and 3 (05-07/06/16), based on three technical replicates taken each day in the morning, noon, and afternoon.

ESM 4

Title: Figure S4. Physiological variables of *Stylophora pistillata* under elevated temperature and reduced pH.

Caption: All graphs show treatment responses to ambient (white) or high temperatures (grey) under ambient pH (left side) or reduced pH conditions (right side) after 2 months. Boxplot depictions as described in Fig. 2. (a) Photosynthetic compensation points (I_k_), (b) Relative initial slopes of rapid light curves (rα), (c) Symbiont chl *a*/chl *c_2_*-ratios, (d) Holobiont areal total chl content, (e) Holobiont respiration, (f) Host O_2_ demand in light (P_gross_/R). Capital letters show significant treatment differences based on Tukey HSD *post hoc* results for significant interactive effects of pH and temperature. Asterisks indicate an overall significant pH effect (Table S1).

ESM 5

Title: Supplementary Methods

NO CAPTION

ESM 6

Title: Table S1. Statistical output of mixed model analysis with indicated effect size for significant factors.

Caption: Variables that are significantly affected by one or more factors are highlighted grey. The type of normalization (per coral surface area; per symbiont cell; per unit protein) is given in brackets. Values for effect size represent mean pairwise relative changes to ambient treatment. Effect sizes for significant main factors are pooled across treatments (e.g. high vs. ambient temperature, irrespective of pH). Asterisks indicate significance at p ≤ 0.05.

ESM 7

Title: Table S2. Summary of replicate-specific turnover values for carbon and nitrogen from NanoSIMS image analysis.

Caption: Regions of interest (ROI) were based on coenosarc cross sections and refer to the oral tissue layer (cf. Fig. 3a-e). Gastrodermis ROIs refer to gastrodermis excluding symbionts, but including host lipids.

ESM 8

Title: Table S3. Statistical ouput for NanoSIMS data.

Caption: Statistical results of pairwise tests (N = 3) for effects of elevated temperature under ambient pH in different regions of interest (ROIs) within oral layers of the coenosarc coral tissue. ROIs with a consistent temperature effect are highlighted (grey). Asterisks indicate significance at p ≤ 0.05.

ESM 9

Title: Table S4. Alkalinity and oxygen saturation in respiration chambers.

Caption: Change in alkalinity and oxygen saturation in respiration chambers used for estimates of calcification, photosynthesis, and holobiont respiration. Since oxygen production/consumption rates were based on a shorter time frames (i.e., the actual linear part of the incubation curve; on average 37±15 min for dark and 37±10 min for light incubation; mean±SD, N=36), we provided an estimate for the maximal oxygen change in the chambers (250-270 mL) based on the total incubation time applied for total alkalinity (TA) measurements (ca. 1-1.5h).

EMS 10

Title: Table S5. Physiological raw data

NO CAPTION

EMS 11

Title: Table S6. Two-way ANOVA outputs testing for a consistent effect of mixing tank for the pH 7.8 treatments for all main physiological variables presented in Fig. 3.

Caption: Triplicate set DEF was tested against GHI across both temperature treatments (cf. Fig. S2). Coral variables that were significantly different between supply tanks are highlighted grey. Asterisks indicate significance at p ≤ 0.05. Note that the borderline significant effect of the amount of host protein (p=0.03) is not unexpected and signifies that replicates G, H, I had on average a slightly larger tissue thickness than D, E, F replicates, which was also consistent across temperatures.
