# Supplementary material for: Common reef-building coral in the Northern Red Sea resistant to elevated temperature and acidification
Source: R Soc Open Sci. 2017 May 17;4(5):170038. doi: 10.1098/rsos.170038 (PMC5451809; doi:10.1098/rsos.170038)
Supplement: Figure S1. Experimental setup and long-term warming trends [file rsos170038supp3.pdf]

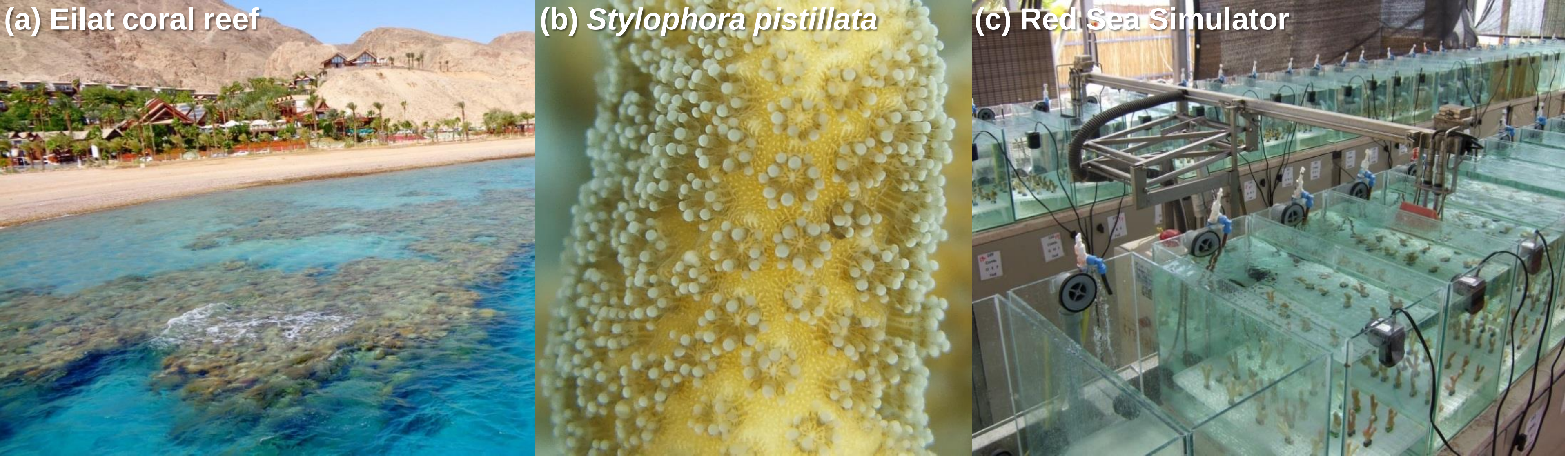

(d) Long-term SST in the GoA (Eilat area) 1988-2016

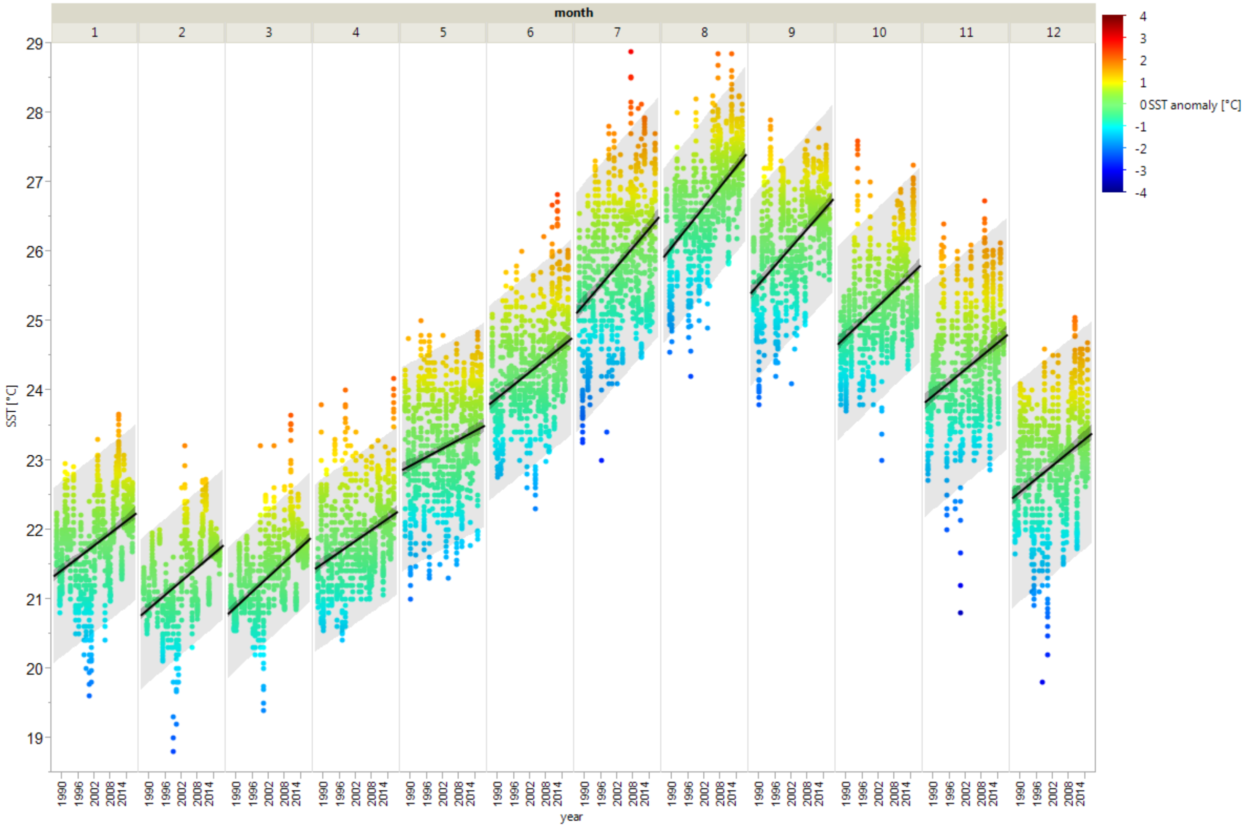

(e) Monthly warming rates 1988-2016 [°C/decade]

Jan	0.30±0.03
Feb	0.33±0.02
Mar	0.36±0.02
Apr	0.27±0.03
May	0.21±0.03
Jun	0.31±0.03
Jul	0.45±0.04
Aug	0.48±0.03
Sep	0.44±0.03
Oct	0.37±0.03
Nov	0.32±0.04
Dec	0.32±0.03
