# Supplementary figures and images for: Common reef-building coral in the Northern Red Sea resistant to elevated temperature and acidification
Source: R Soc Open Sci. 2017 May 17;4(5):170038. doi: 10.1098/rsos.170038 (PMC5451809; doi:10.1098/rsos.170038)

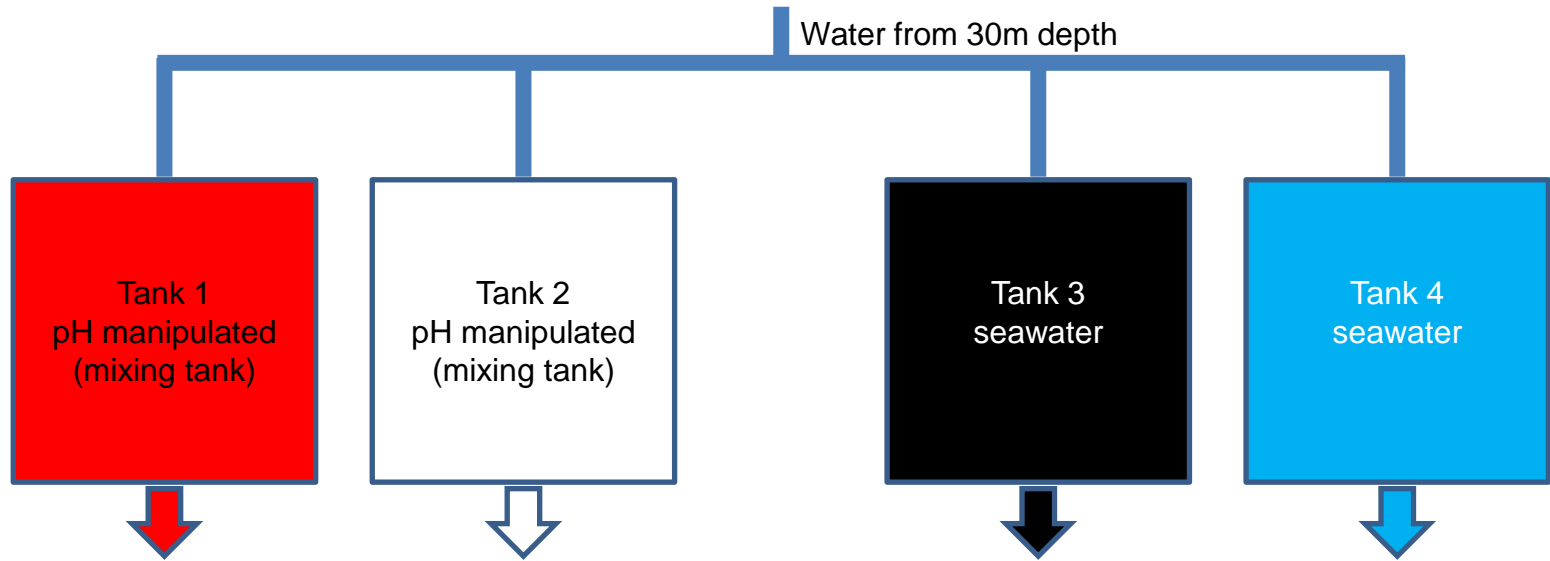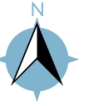

Temperature manipulated; experimental aquaria

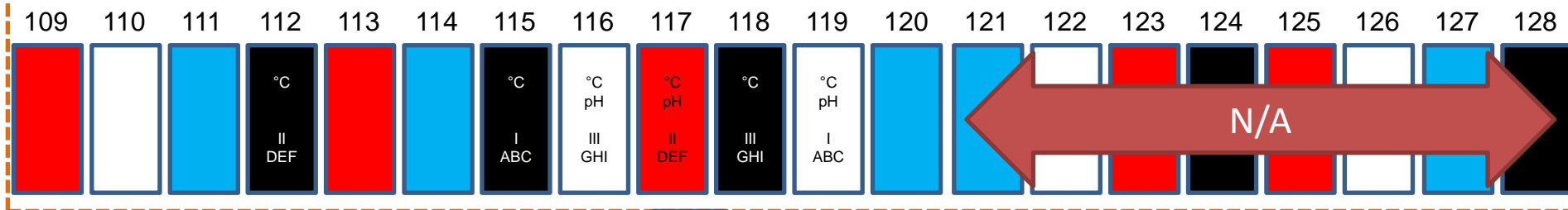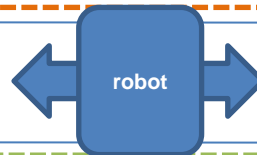

Ambient temperature; experimental aquaria

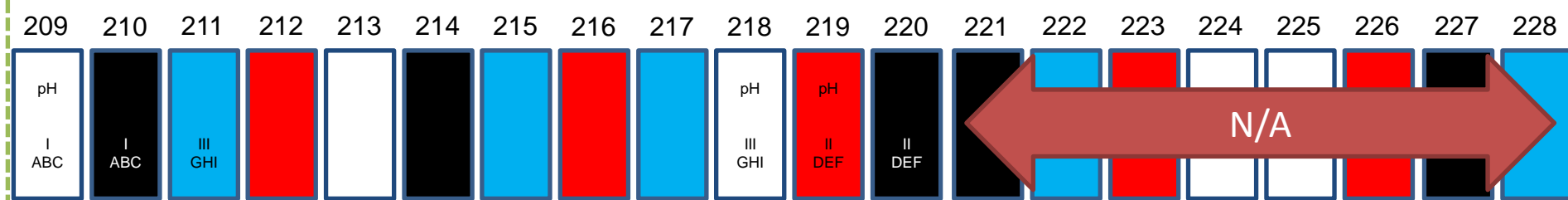

Supplement: Figure S2. Schematic of the experimental layout in the IUI's Red Sea Simulator aquaria array [file rsos170038supp4.pdf]

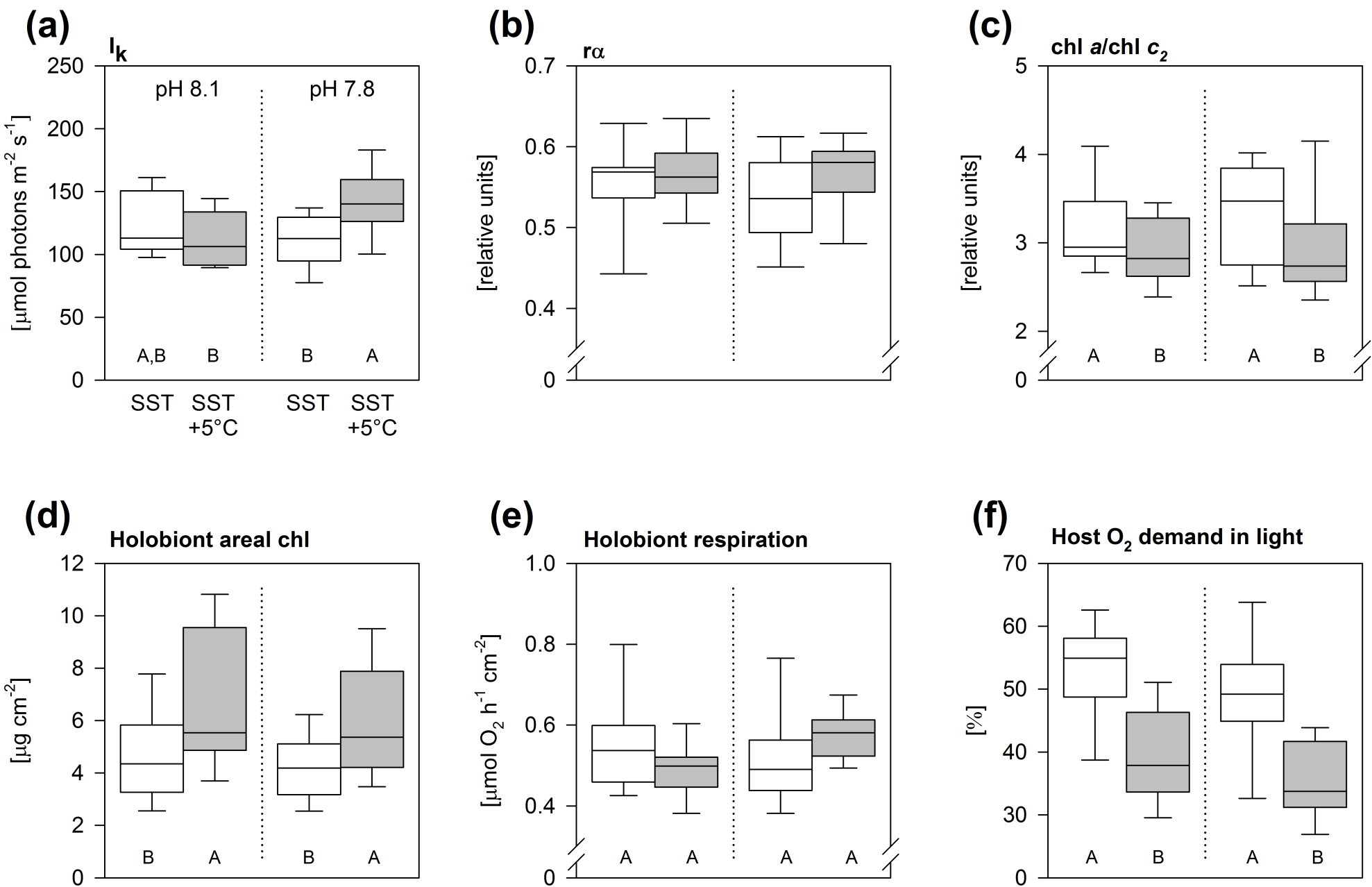

Supplement: Figure S4. Physiological variables of Stylophora pistillata under elevated temperature and reduced pH [file rsos170038supp6.pdf]
