# Supplementary material for: Common reef-building coral in the Northern Red Sea resistant to elevated temperature and acidification
Source: R Soc Open Sci. 2017 May 17;4(5):170038. doi: 10.1098/rsos.170038 (PMC5451809; doi:10.1098/rsos.170038)
Supplement: Figure S3. Monitoring data of experimental setup [file rsos170038supp5.pdf]

(a)

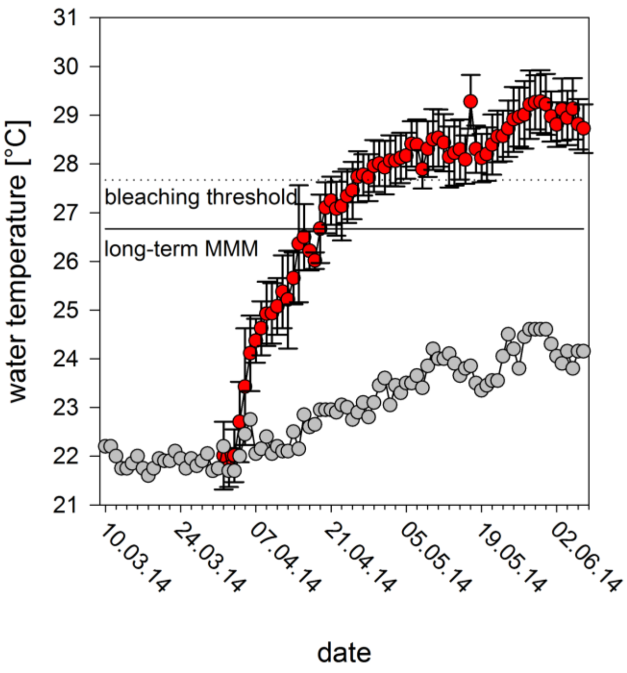

(b)

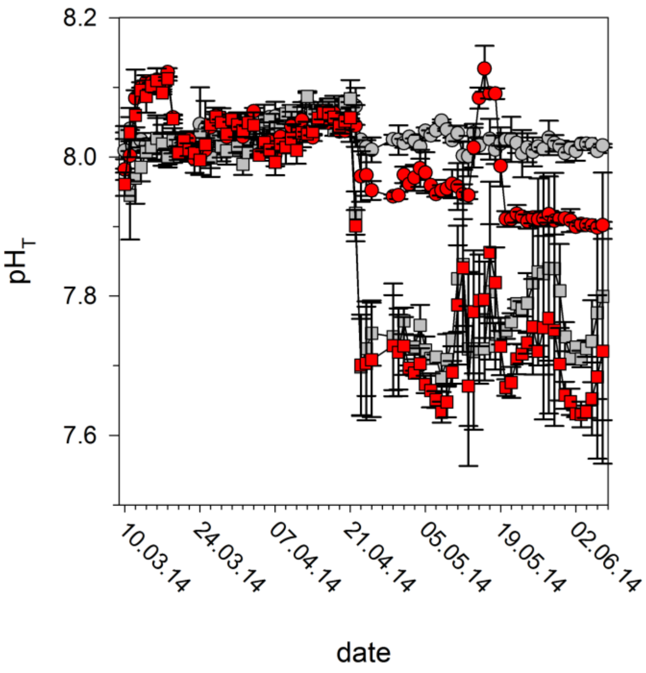

(c)

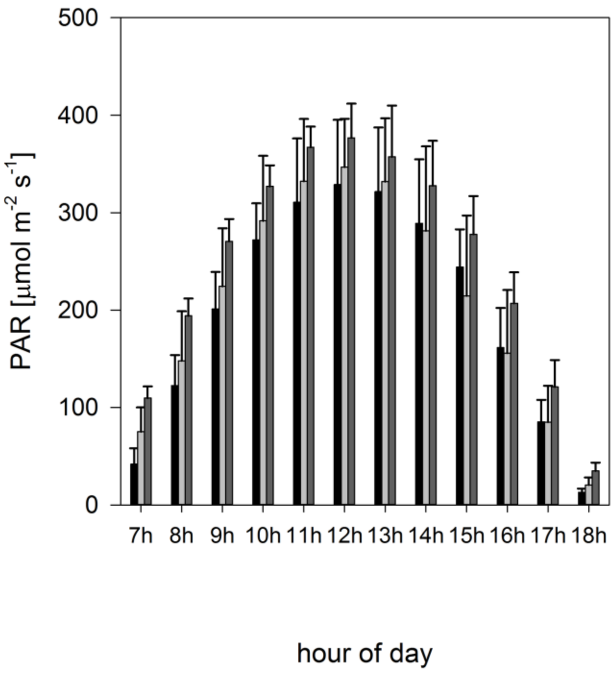

(d)

pH_T	SD	Temp [°C]	Time period	TA [µmol kg^{-1}]	SD	DIC [µmol kg^{-1}]	SD	pCO_2 [µatm]	SD	$CO_{2(aq)}$ [µmol kg^{-1}]	SD	HCO_3^- [µmol kg^{-1}]	SD	CO_3^{2-} [µmol kg^{-1}]	SD	Ω_{arag}	SD
8.02	0.00	23	27-29/04/14	2499.67	2.99	2183.93	1.21	466.77	3.19	13.54	0.09	1948.03	1.07	222.36	1.39	3.36	0.02
8.02	0.01	23	18-20/05/14	2501.21	1.45	2188.50	3.84	473.77	9.29	13.75	0.27	1954.23	6.35	220.51	2.88	3.33	0.04
8.01	0.00	24	05-07/06/14	2493.47	5.67	2176.00	8.32	479.63	7.92	13.56	0.22	1938.83	9.53	223.61	1.46	3.39	0.02
7.95	0.02	28	27-29/04/14	2498.58	6.53	2188.20	6.98	581.47	26.99	14.91	0.69	1952.37	13.34	220.90	7.52	3.41	0.12
8.00	0.09	28	18-20/05/14	2500.46	2.01	2160.73	61.22	526.23	131.95	13.49	3.38	1907.70	95.82	239.52	37.98	3.70	0.59
7.90	0.00	29	05-07/06/14	2489.17	10.55	2205.50	10.66	675.60	6.09	16.92	0.15	1984.27	10.2	204.28	0.45	3.17	0.01
7.74	0.02	23	27-29/04/14	2501.70	3.33	2338.70	13.60	999.17	59.75	28.99	1.73	2179.43	17.55	130.26	5.70	1.97	0.09
7.74	0.01	23	18-20/05/14	2503.68	3.17	2341.73	7.34	1005.1	29.82	29.16	0.87	2182.87	9.12	129.71	2.79	1.96	0.04
7.77	0.03	24	05-07/06/14	2496.89	0.43	2315.03	15.30	933.90	80.54	26.41	2.28	2147.23	22.15	141.38	9.08	2.15	0.14
7.73	0.01	28	27-29/04/14	2504.85	3.74	2322.30	5.15	1083.77	15.83	27.79	0.41	2150.83	5.85	143.69	1.43	2.22	0.02
7.74	0.08	28	18-20/05/14	2502.13	4.21	2312.20	41.80	1058.63	208.47	27.14	5.34	2136.90	58.59	148.16	22.12	2.29	0.34
7.69	0.03	29	05-07/06/14	2492.71	1.78	2324.37	15.23	1208.60	107.14	30.27	2.68	2158.43	21.94	135.68	9.35	2.10	0.15
