# Supplementary material for: Common reef-building coral in the Northern Red Sea resistant to elevated temperature and acidification
Source: R Soc Open Sci. 2017 May 17;4(5):170038. doi: 10.1098/rsos.170038 (PMC5451809; doi:10.1098/rsos.170038)
Supplement: Table S1. Statistical output of mixed model analysis with indicated effect size for significant factors [file rsos170038supp7.docx]

Table S1. Statistical output of mixed model analysis with indicated effect size for significant factors. Variables that are significantly affected by one or more factors are highlighted grey. The type of normalization (per coral surface area; per symbiont cell; per unit protein) is given in brackets. Values for effect size represent the mean of the pairwise relative change of each replicate relative to the ambient treatment. Effect sizes for significant main factors are pooled across treatments (e.g. high vs. ambient temperature, irrespective of pH). Asterisks indicate significance at p ≤ 0.05.

|  | **Temperature** | **Effect size** | **pH** | **Effect size** | **Temperature x pH** | **Effect size** | **Replicate tank** | **Relative variance component** |
| --- | --- | --- | --- | --- | --- | --- | --- | --- |
| F_v_/F_m_ | *F*_1,24_ = 5.854,  p = 0.0235* | +5±4% | *F*_1,24_ = 2.8652,  p = 0.1035 |  | *F*_1,24_ = 6.5001,  p = 0.0176* | +6±3% | *F*_2,6_ = 1.2064,  p = 0.3628 | 46.8% |
| rETR_max_ | *F*_1,24_ = 1.2292,  p = 0.1907 |  | *F*_1,24_ = 3.6249,  p = 0.0690 |  | *F*_1,24_ = 12.9225,  p = 0.0015* | +19±30% | *F*_2,6_ = 2.2119,  p = 0.1907 | 38.6% |
| rα | *F*_1,25_ = 1.9837,  p = 0.1713 |  | *F*_1,25_ = 0.4480,  p = 0.5094 |  | N/A |  | *F*_2,6_ = 1.4600,  p = 0.3043 | 33.2% |
| I_k_ | *F*_1,24_ = 2.6025,  p = 0.1198 |  | *F*_1,24_ = 2.9244,  p = 0.1001 |  | *F*_1,24_ = 14.8554,  p = 0.0008* | +16±24% | *F*_2,6_ = 1.8553,  p = 0.2359 | 49.2% |
| Symbiont density | *F*_1,25_ = 0.0525,  p = 0.8207 |  | *F*_1,25_ = 0.0002,  p = 0.9900 |  | N/A |  | *F*_2,6_ = 0.1129,  p = 0.8951 | 76.7% |
| Total Chl (cell) | *F*_1,25_ = 77.2526,  p < 0.0001* | +45±18% | *F*_1,25_ = 4.3735,  p = 0.0468* | -9±9% | N/A |  | *F*_2,6_ = 10.8541,  p = 0.0102* | 0.0% |
| Chl *a* (cell) | *F*_1,25_ = 70.2747,  p < 0.0001* | +41±18% | *F*_1,25_ = 4.8427,  p = 0.0372* | -9±9% | N/A |  | *F*_2,6_ = 6.2986,  p = 0.0336* | 0.0% |
| Chl *c* (cell) | *F*_1,25_ = 68.4197,  p < 0.0001* | +56±21% | *F*_1,25_ = 4.8858,  p = 0.0365* | -11±12% | N/A |  | *F*_2,6_ = 16.4496,  p = 0.0037* | 13.6% |
| Chl *a*/chl *c_2_* | *F*_1,25_ = 10.9676,  p = 0.0041* | -10±10% | *F*_1,25_ = 0.7145,  p = 0.4060 |  | N/A |  | *F*_2,6_ = 15.7475,  p = 0.0041* | 24.5% |
| Total Chl (surface) | *F*_1,25_ = 26.9715,  p < 0.0001* | +44±36% | *F*_1,25_ = 1.6660,  p = 0.2086 |  | N/A |  | *F*_2,6_ = 0.8838,  p = 0.4609 | 74.1% |
| P_gross_ (surface) | *F*_1,24_ = 7.3749,  p = 0.0121* | +39±23% | *F*_1,24_ = 0.0054,  p = 0.9422 |  | *F*_1,24_ = 18.2813,  p = 0.0003* | +61±34% | *F*_2,6_ = 1.0685,  p = 0.4009 | 79.3% |
| P_net_ (surface) | *F*_1,24_ = 17.5486,  p = 0.0003* | +83±44% | *F*_1,24_ = 0.4495,  p = 0.5090 |  | *F*_1,24_ = 6.6075,  p = 0.0168* | +129±82% | *F*_2,6_ = 1.6447,  p = 0.2694 | 68.2% |
| Respiration (surface) | *F*_1,24_ = 2.5806,  p = 0.1213 |  | *F*_1,24_ = 0.8098,  p = 0.3771 |  | *F*_1,24_ = 4.8974,  p = 0.0367* | +7±25% | *F*_2,6_ = 0.2390,  p = 0.7946 | 42.9% |
| P_gross_ (Chl) | *F*_1,25_ = 0.3673,  p = 0.5499 |  | *F*_1,25_ = 10.2562,  p = 0.0037* | +27±23% | N/A |  | *F*_2,6_ = 2.5871,  p = 0.1548 | 23.6% |
| P_net_ (Chl) | *F*_1,25_ = 5.1081,  p = 0.0328* | +29±42% | *F*_1,25_ = 10.4800,  p = 0.0034* | +40±37% | N/A |  | *F*_2,6_ = 2.3524,  p = 0.1761 | 6.3% |
| Light R:P_gross_ | *F*_1,25_ = 39.3848,  p < 0.0001* | -25±13% | *F*_1,25_ = 3.6185,  p = 0.0687 |  | N/A |  | *F*_2,6_ = 2.5341,  p = 0.1593 | 33.7% |
| daily P_gross_:R | *F*_1,25_ = 39.2618,  p < 0.0001* | +39±27% | *F*_1,25_ = 4.8050,  p = 0.0379* | +12±19% | N/A |  | *F*_2,6_ = 1.7341,  p = 0.2545 | 48.8% |
| Light calcification (surface) | *F*_1,25_ = 4.0142,  p = 0.0561 |  | *F*_1,25_ = 0.6671,  p = 0.4218 |  | N/A |  | *F*_2,6_ = 0.4120,  p = 0.6797 | 21.5% |
| Dark calcification (surface) | *F*_1,25_ = 2.8891,  p = 0.1016 |  | *F*_1,25_ = 0.9172,  p = 0.3474 |  | N/A |  | *F*_2,6_ = 0.4759,  p = 0.6429 | 12.0% |
| Symbiont SOD (protein) | *F*_1,25_ = 0.5078,  p = 0.4827 |  | *F*_1,25_ = 0.7042,  p = 0.4093 |  | N/A |  | *F*_2,6_ = 0.3715,  p = 0.7045 | 53.8% |
| Symbiont KatG (protein) | *F*_1,25_ = 1.9111,  p = 0.1791 |  | *F*_1,25_ = 0.6453,  p = 0.4249 |  | N/A |  | *F*_2,6_ = 0.8037,  p = 0.4906 | 58.4% |
| Symbiont protein (cell) | *F*_1,25_ = 2.9638,  p = 0.0975 |  | *F*_1,25_ = 0.0014,  p = 0.9702 |  | N/A |  | *F*_2,6_ = 0.1103,  p = 0.8973 | 90.0% |
| Symbiont carbohydrate (cell) | *F*_1,25_ = 0.0055,  p = 0.9415 |  | *F*_1,25_ = 1.0506,  p = 0.3152 |  | N/A |  | *F*_2,6_ = 0.0009,  p = 0.9991 | 85.5% |
| Host SOD (protein) | *F*_1,25_ = 0.3903,  p = 0.5378 |  | *F*_1,25_ = 2.0525,  p = 0.1643 |  | N/A |  | *F*_2,6_ = 2.6124,  p = 0.1527 | 12.7% |
| Host CAT (protein) | *F*_1,25_ = 5.6520,  p = 0.0254* | +14±13% | *F*_1,25_ = 1.1917,  p = 0.2854 |  | N/A |  | *F*_2,6_ = 0.4704,  p = 0.6460 | 2.9% |
| Host protein (surface) | *F*_1,25_ = 0.9077,  p = 0.3498 |  | *F*_1,25_ = 0.0622,  p = 0.8050 |  | N/A |  | *F*_2,6_ = 3.4819,  p = 0.0991 | 26.3% |
| Host carbohydrate (surface) | *F*_1,25_ = 0.2195,  p = 0.6435 |  | *F*_1,25_ = 0.6056,  p = 0.4437 |  | N/A |  | *F*_2,6_ = 1.9032,  p = 0.2290 | 25.0% |
