# Supplementary material for: Common reef-building coral in the Northern Red Sea resistant to elevated temperature and acidification
Source: R Soc Open Sci. 2017 May 17;4(5):170038. doi: 10.1098/rsos.170038 (PMC5451809; doi:10.1098/rsos.170038)
Supplement: Table S2. Summary of replicate-specific turnover values for carbon and nitrogen from NanoSIMS image analysis [file rsos170038supp8.docx]

Table S2. Summary of replicate-specific turnover values for carbon and nitrogen from NanoSIMS image analysis. Regions of interest (ROI) were based on coenosarc cross sections and refer to the oral tissue layer (cf. Fig. 4a-e). Gastrodermis ROIs refer to gastrodermis excluding symbionts, but including host lipids.

|  |  |  | **^13^C turnover [%]** | | **^15^N turnover [%]** | |  |  |  |
| --- | --- | --- | --- | --- | --- | --- | --- | --- | --- |
| **temperature** | **ROI** | **replicate** | **mean** | **SE** | **mean** | **SE** | **ROI diameter ±SE [µm]** | **ROI area ±SE [µm^2^]** | **N** |
| ambient | Symbiont | A | 5.3400 | 0.2158 | 2.2901 | 0.0738 | 7.8±0.3 |  | 52 |
| ambient | Symbiont | G | 5.9558 | 0.3149 | 2.0309 | 0.0582 | 6.7±0.2 |  | 50 |
| ambient | Symbiont | I | 5.2209 | 0.2241 | 1.5642 | 0.0685 | 7.3±0.2 |  | 57 |
| ambient | Gastrodermis | A | 1.5701 | 0.1957 | 0.1733 | 0.0171 |  | 354.7±30.1 | 11 |
| ambient | Gastrodermis | G | 1.6326 | 0.0908 | 0.2286 | 0.0176 |  | 280.4±18.7 | 10 |
| ambient | Gastrodermis | I | 0.9182 | 0.1378 | 0.0990 | 0.0160 |  | 349.2±28.7 | 13 |
| ambient | Host lipid | A | 8.0961 | 0.2871 | 0.2545 | 0.0239 | 2.7±0.2 |  | 61 |
| ambient | Host lipid | G | 7.7857 | 0.2193 | 0.2643 | 0.0243 | 2.5±0.2 |  | 57 |
| ambient | Host lipid | I | 8.1860 | 0.3705 | 0.2537 | 0.0336 | 2.7±0.2 |  | 44 |
| ambient | Epidermis | A | 0.0164 | 0.0056 | 0.0106 | 0.0015 |  | 586.2±42.4 | 11 |
| ambient | Epidermis | G | 0.0531 | 0.0033 | 0.0164 | 0.0005 |  | 391.6±29.1 | 10 |
| ambient | Epidermis | I | 0.0447 | 0.0058 | 0.0103 | 0.0012 |  | 508.6±57.1 | 13 |
| high | Symbiont | A | 5.3884 | 0.2693 | 1.9603 | 0.0494 | 8.1±0.3 |  | 47 |
| high | Symbiont | G | 4.1101 | 0.2121 | 1.4193 | 0.0455 | 7.6±0.3 |  | 56 |
| high | Symbiont | I | 4.1919 | 0.2116 | 1.9761 | 0.0644 | 6.9±0.3 |  | 47 |
| high | Gastrodermis | A | 1.5517 | 0.1656 | 0.1488 | 0.0085 |  | 282.2±17.5 | 14 |
| high | Gastrodermis | G | 0.5637 | 0.0534 | 0.0534 | 0.0061 |  | 293.9±13.3 | 22 |
| high | Gastrodermis | I | 0.6657 | 0.1052 | 0.0844 | 0.0103 |  | 284.2±18.9 | 16 |
| high | Host lipid | A | 9.1415 | 0.3552 | 0.2410 | 0.0246 | 3.0±0.2 |  | 46 |
| high | Host lipid | G | 7.2823 | 0.3322 | 0.1567 | 0.0163 | 2.2±0.1 |  | 39 |
| high | Host lipid | I | 5.5690 | 0.2101 | 0.2093 | 0.0283 | 2.4±0.2 |  | 35 |
| high | Epidermis | A | 0.0326 | 0.0026 | 0.0060 | 0.0008 |  | 651.5±37.6 | 14 |
| high | Epidermis | G | 0.0530 | 0.0033 | 0.0078 | 0.0005 |  | 588.6±17.0 | 22 |
| high | Epidermis | I | 0.0242 | 0.0051 | 0.0089 | 0.0009 |  | 557.9±26.6 | 16 |
