# Supplementary material for: Common reef-building coral in the Northern Red Sea resistant to elevated temperature and acidification
Source: R Soc Open Sci. 2017 May 17;4(5):170038. doi: 10.1098/rsos.170038 (PMC5451809; doi:10.1098/rsos.170038)
Supplement: Table S3. Statistical ouput for NanoSIMS data [file rsos170038supp9.docx]

Table S3. Statistical output for NanoSIMS data. Statistical results of pairwise tests (N = 3) for effects of elevated temperature under ambient pH in different regions of interest (ROIs) within oral layers of the coenosarc coral tissue. ROIs with a consistent temperature effect are highlighted (grey). Asterisks indicate significance at p ≤ 0.05.

| **isotope** | **ROI** | **temperature** | **replicate** | **temperature x replicate** |
| --- | --- | --- | --- | --- |
| δ^13^C | Symbiont | F_1,305_ = 22.16,  p < 0.0001* | F_2,304_ = 3.16,  p = 0.0439* | F_5,302_ = 7.28,  p = 0.0008* |
|  | Gastrodermis | F_1,82_ = 17.22,  p < 0.0001* | F_2,81_ = 16.53,  p < 0.0001* | F_5,79_ = 9.44,  p = 0.0002* |
|  | Host lipid bodies | F_1,279_ = 3.74,  p = 0.0542 | F_2,278_ = 11.50,  p < 0.0001* | F_5,276_ = 17.36,  p < 0.0001* |
|  | Epidermis | F_1,82_ = 0.36,  p = 0.5513 | F_2,81_ = 17.95,  p < 0.0001* | F_5,79_ = 8.22,  p = 0.0006* |
| δ^15^N | Symbiont | F_1,305_ = 10.03,  p = 0.0017* | F_2,304_ = 22.69,  p < 0.0001* | F_5,302_ = 37.57,  p < 0.0001* |
|  | Gastrodermis | F_1,82_ = 28.37,  p < 0.0001* | F_2,81_ = 11.95,  p < 0.0001* | F_5,79_ = 27.17,  p < 0.0001* |
|  | Host lipid bodies | F_1,276_ = 5.08,  p = 0.0251* | F_2,275_ = 0.60,  p = 0.5502 | F_5,273_ = 2.07,  p = 0.1285 |
|  | Epidermis | F_1,82_ = 30.77,  p < 0.0001* | F_2,81_ = 6.51,  p = 0.0024* | F_5,79_ = 5.27,  p = 0.0071* |
