# Supplementary material for: Common reef-building coral in the Northern Red Sea resistant to elevated temperature and acidification
Source: R Soc Open Sci. 2017 May 17;4(5):170038. doi: 10.1098/rsos.170038 (PMC5451809; doi:10.1098/rsos.170038)
Supplement: Table S4. Alkalinity and oxygen saturation in respiration chambers [file rsos170038supp10.docx]

Table S4. Alkalinity and oxygen saturation in respiration chambers. Change in alkalinity and oxygen saturation in respiration chambers used for estimates of calcification, photosynthesis, and holobiont respiration. Since oxygen production/consumption rates were based on a shorter time frames (i.e., the actual linear part of the incubation curve; on average 37±15 min for dark and 37±10 min for light incubation; mean±SD, N=36), we provided an estimate for the maximal oxygen change in the chambers (250-270 mL) based on the total incubation time applied for total alkalinity (TA) measurements (ca. 1-1.5h).

|  |  |  |  | **DARK INCUBATION** | | | | **LIGHT INCUBATION** | | |
| --- | --- | --- | --- | --- | --- | --- | --- | --- | --- | --- |
| **pH_NBS_** | **Temp [°C]** | **Repl** | **Initial TA [µmol kg^-1^]** | **Incubation time [h]** | **final TA**  **[µmol kg^-1^]** | **max oxygen change [%]** | **Incubation time [h]** | | **final TA**  **[µmol kg^-1^]** | **max oxygen change [%]** |
| 8.1 | ambient | A | 2487.90 | 1.05 | 2474.60 | -17 | 1.00 | | 2450.10 | +15 |
| 8.1 | ambient | B | 2488.60 | 1.08 | 2481.20 | -20 | 0.92 | | 2460.20 | +33 |
| 8.1 | ambient | C | 2490.60 | 1.05 | 2469.30 | -21 | 1.12 | | 2449.25 | +32 |
| 8.1 | ambient | D | 2494.15 | 0.98 | 2479.25 | -23 | 1.05 | | 2467.65 | +16 |
| 8.1 | ambient | E | 2501.25 | 1.15 | 2470.65 | -17 | 0.95 | | 2448.00 | +12 |
| 8.1 | ambient | F | 2504.15 | 1.08 | 2493.75 | -19 | 1.03 | | 2481.25 | +12 |
| 8.1 | ambient | G | 2501.40 | 1.12 | 2454.75 | -14 | 1.25 | | 2470.30 | +17 |
| 8.1 | ambient | H | 2475.90 | 0.98 | 2464.20 | -22 | 1.05 | | 2439.60 | +23 |
| 8.1 | ambient | I | 2497.25 | 1.08 | 2483.75 | -15 | 1.00 | | 2468.25 | +12 |
| 8.1 | elevated | A | 2488.45 | 1.15 | 2464.85 | -19 | 0.87 | | 2451.25 | +29 |
| 8.1 | elevated | B | 2492.40 | 1.10 | 2482.75 | -17 | 0.98 | | 2470.00 | +43 |
| 8.1 | elevated | C | 2489.90 | 1.12 | 2483.65 | -18 | 1.13 | | 2467.40 | +48 |
| 8.1 | elevated | D | 2493.65 | 1.10 | 2466.85 | -12 | 1.02 | | 2474.30 | +30 |
| 8.1 | elevated | E | 2498.00 | 1.15 | 2483.05 | -11 | 1.25 | | 2474.65 | +32 |
| 8.1 | elevated | F | 2505.75 | 1.03 | 2495.60 | -20 | 0.90 | | 2482.95 | +22 |
| 8.1 | elevated | G | 2481.80 | 1.13 | 2492.85 | -13 | 1.05 | | 2449.15 | +38 |
| 8.1 | elevated | H | 2476.40 | 1.08 | 2470.30 | -20 | 1.05 | | 2453.95 | +28 |
| 8.1 | elevated | I | 2476.15 | 1.23 | 2465.00 | -18 | 1.05 | | 2459.85 | +20 |
| 7.8 | ambient | A | 2495.20 | 1.18 | 2472.30 | -17 | 1.03 | | 2456.60 | +15 |
| 7.8 | ambient | B | 2502.70 | 1.17 | 2463.65 | -16 | 0.85 | | 2451.75 | +30 |
| 7.8 | ambient | C | 2491.35 | 1.12 | 2472.95 | -14 | 1.10 | | 2460.50 | +26 |
| 7.8 | ambient | D | 2494.75 | 1.17 | 2463.75 | -32 | 0.82 | | 2469.60 | +12 |
| 7.8 | ambient | E | 2498.35 | 1.15 | 2471.65 | -10 | 1.12 | | 2466.70 | +13 |
| 7.8 | ambient | F | 2498.00 | 1.17 | 2494.35 | -17 | 0.98 | | 2480.95 | +18 |
| 7.8 | ambient | G | 2494.60 | 1.20 | 2450.05 | -42 | 1.10 | | 2457.80 | +27 |
| 7.8 | ambient | H | 2500.75 | 1.13 | 2462.30 | -27 | 0.98 | | 2439.10 | +25 |
| 7.8 | ambient | I | 2496.35 | 1.27 | 2477.30 | -23 | 0.77 | | 2479.10 | +15 |
| 7.8 | elevated | A | 2486.65 | 1.22 | 2488.55 | -32 | 1.05 | | 2453.65 | +44 |
| 7.8 | elevated | B | 2493.00 | 1.22 | 2472.45 | -19 | 0.92 | | 2459.05 | +53 |
| 7.8 | elevated | C | 2492.45 | 1.17 | 2475.00 | -19 | 1.08 | | 2467.85 | +52 |
| 7.8 | elevated | D | 2490.75 | 1.28 | 2430.75 | -29 | 0.90 | | 2445.65 | +48 |
| 7.8 | elevated | E | 2493.40 | 1.35 | 2474.05 | -14 | 1.18 | | 2479.00 | +28 |
| 7.8 | elevated | F | 2498.15 | 1.17 | 2495.20 | -7 | 0.95 | | 2487.45 | +22 |
| 7.8 | elevated | G | 2495.05 | 1.13 | 2500.85 | -23 | 0.97 | | 2461.65 | +45 |
| 7.8 | elevated | H | 2491.40 | 1.15 | 2452.55 | -19 | 1.00 | | 2442.80 | +36 |
| 7.8 | elevated | I | 2493.55 | 1.45 | 2484.40 | -32 | 0.88 | | 2489.00 | +27 |
