# Supplementary material for: Common reef-building coral in the Northern Red Sea resistant to elevated temperature and acidification
Source: R Soc Open Sci. 2017 May 17;4(5):170038. doi: 10.1098/rsos.170038 (PMC5451809; doi:10.1098/rsos.170038)
Supplement: Table S6. Two-way ANOVA outputs testing for a consistent effect of mixing tank for the pH 7.8 treatments for all main physiological variables presented in Fig. 3 [file rsos170038supp12.docx]

Table S6. Two-way ANOVA outputs testing for a consistent effect of mixing tank for the pH 7.8 treatments for all main physiological variables presented in Fig. 3. Triplicate set DEF was tested against GHI across both temperature treatments (cf. Fig. S2). Coral variables that were significantly different between supply tanks are highlighted grey. Asterisks indicate significance at p ≤ 0.05. Note that the borderline significant effect of the amount of host protein (p=0.03) is not unexpected and signifies that replicates G, H, I had on average a slightly larger tissue thickness than D, E, F replicates, which was also consistent across temperatures.

| **Variable** | **Temp** | **Mixing tank** | **Temp x Mixing tank** |
| --- | --- | --- | --- |
| density | F_1,11_ = 0.5280;  p = 0.2226 | F_1,11_ = 1.7487,  p = 0.4882 | F_1,9_ = 0.1886,  p = 0.6756 |
| Chl content | F_1,11_ = 18.4990;  p = 0.0026* | F_1,11_ = 0.0201,  p = 0.8909 | F_1,9_ = 1.5780,  p = 0.2445 |
| Fv/Fm | F_1,11_ = 13.4837;  p = 0.0063* | F_1,11_ = 0.1824,  p = 0.6806 | F_1,9_ = 0.0469,  p = 0.8340 |
| rETRmax | F_1,11_ = 3.7248;  p = 0.0897 | F_1,11_ = 0.8097,  p = 0.3945 | F_1,9_ = 0.5911,  p = 0.4641 |
| Net O2 | F_1,11_ = 63.1374;  p < 0.0001* | F_1,11_ = 0.4644,  p = 0.5148 | F_1,9_ = 1.7776,  p = 0.2192 |
| Gross O2/Chl | F_1,11_ = 0.3152;  p = 0.5899 | F_1,11_ = 1.8779,  p = 0.2078 | F_1,9_ = 0.3222,  p = 0.5858 |
| Daily P_gross_:R | F_1,11_ = 19.0103;  p = 0.0024* | F_1,11_ = 0.0035,  p = 0.9541 | F_1,9_ = 0.0206,  p = 0.8895 |
| Dark Calcification | F_1,11_ = 0.0671;  p = 0.8022 | F_1,11_ = 0.0123,  p = 0.9143 | F_1,9_ = 0.2303,  p = 0.6442 |
| Light Calcification | F_1,11_ = 0.0008;  p = 0.9785 | F_1,11_ = 0.0029,  p = 0.9583 | F_1,9_ < 0.0001,  p = 0.9980 |
| Host SOD | F_1,11_ = 1.7512;  p = 0.2223 | F_1,11_ = 0.0642,  p = 0.8064 | F_1,9_ = 0.6835,  p = 0.4324 |
| Symbiont SOD | F_1,11_ = 0.6511;  p = 0.4431 | F_1,11_ = 0.2809,  p = 0.6105 | F_1,9_ = 0.0547,  p = 0.8210 |
| Host CAT | F_1,11_ = 4.3672;  p = 0.0700 | F_1,11_ = 0.9265,  p = 0.3640 | F_1,9_ = 0.3233,  p = 0.5852 |
| Symbiont CAT | F_1,11_ = 0.1236;  p = 0.7342 | F_1,11_ = 1.6382,  p = 0.2364 | F_1,9_ = 0.3265,  p = 0.5834 |
| Host protein | F_1,11_ = 0.0355;  p = 0.8553 | F_1,11_ = 6.7972,  p = 0.0313* | F_1,9_ = 0.5418,  p = 0.4827 |
| Symbiont protein | F_1,11_ = 0.0079;  p = 0.9313 | F_1,11_ = 0.3864,  p = 0.5515 | F_1,9_ = 0.2267,  p = 0.6467 |
| Host carbohydrates | F_1,11_ = 0.3974;  p = 0.5460 | F_1,11_ = 1.4911,  p = 0.2568 | F_1,9_ = 0.6722,  p = 0.4360 |
| Symbiont carbohydrates | F_1,11_ = 0.0067;  p = 0.9366 | F_1,11_ = 0.0280,  p = 0.8712 | F_1,9_ = 0.0338,  p = 0.8588 |
